# Leveraging Synthetic Clinical Data for Validation and Operational Readiness in Clinical Trials

**DOI:** 10.3390/healthcare14172870

**Published:** 2026-09-07

**Authors:** Szymon Musik, Jacek Zalewski, Julia Jurkowska, Weronika Pędzimąż, Joanna Sasin-Kurowska, Mariusz Panczyk

**Affiliations:** 1 Department of Education and Research in Health Sciences, Faculty of Health Sciences, Medical University of Warsaw, 00-581 Warsaw, Poland; mariusz.panczyk@wum.edu.pl; 2 Clinical Data & Insights, Biopharmaceutical Clinical Operations, R&D, AstraZeneca, 02-676 Warsaw, Poland

**Keywords:** clinical data management, synthetic data, Bayesian network, clinical data science, clinical research

## Abstract

**Background/Objectives**: Obtaining timely access to detailed clinical trial data is not always straightforward. Privacy requirements, governance processes, and study-specific eCRF configurations can delay access, particularly during study start-up, when teams need realistic data to develop and test validation rules, reporting pipelines, and centralized monitoring tools. **Methods**: We developed SYNDATA, a modular framework that generates synthetic clinical trial datasets conforming to a target electronic case report form (eCRF). The framework combines study metadata from the Medidata Rave Architect Loader Spreadsheet (ALS) with selected empirical patterns learned from a closely matched reference study. It constructs patient-specific timelines from the ALS visit matrix, generates module-specific records using Bayesian networks for selected categorical dependencies and density-based methods for numeric and temporal variables, and applies postprocessing for counters, dictionary coding, and conditional missingness. A configurable Noise Tool injects controlled and reproducible data defects, including timeline inconsistencies, visit-window violations, numeric threshold exceedances, randomization or eligibility conflicts, and structural collisions, to stress-test downstream validation logic. **Results**: Synthetic and source data were compared descriptively using Jensen–Shannon distance, Cramér’s V, and representative plots across selected domains. Several binary operational fields showed close descriptive agreement, whereas agreement was weaker for some more complex, multi-category safety- and medication-related variables. The evaluation covered a representative subset and does not establish uniform fidelity, formal statistical equivalence, or clinical validity across all generated domains. **Conclusions**: SYNDATA supports reproducible generation of ALS/eCRF-conformant datasets for operational validation, reporting development, and workflow testing before real trial data are available. Its demonstrated value is limited to the evaluated operational use case; fidelity is variable-specific, and more complex domains require further development and validation.

## 1. Introduction

Timely and reliable access to granular clinical data is essential for evidence generation, comparative effectiveness research, and methods development in healthcare. Access is often constrained by privacy requirements [1], lengthy governance processes, and heterogeneity in electronic case report form (eCRF) implementations across trials and registries. These constraints slow hypothesis testing, limit reproducibility, and impede rapid iteration during study design and analytics development [2]. Synthetic datasets are designed to reproduce selected statistical characteristics and relationships observed in source data while reducing direct reliance on identifiable records. However, synthetic generation does not inherently guarantee anonymization or the absence of disclosure risk: privacy protection depends on the generation method, source data, release setting, and threat model [1,3,4]. Within clinical trials, producing data that are both ALS/eCRF-conformant and analytically credible requires combining structured study specifications with selected empirical patterns observed in operational data.

In Medidata Rave, study design and data collection are codified through the Architect Loader Spreadsheet (ALS), which defines forms, fields, visit folders, and scheduling windows. The ALS provides a robust scaffold for reproducing a target study’s structure and visit chronology. It does not, however, encode empirical distributions, dependencies among selected fields, or conditional missingness patterns, which may influence downstream analyses. Pairing the ALS with a closely related reference study provides a way to incorporate these empirical features while maintaining conformance to the target workflow and operational cadence. The framework presented here uses this pairing to generate module-specific synthetic datasets aligned with clinical workflows, including dictionary-coded variables and selected temporal relationships across visits and events.

The broader context for this work is the growing use of external data and synthetic controls in clinical evaluation, especially where randomized controls are infeasible or unethical. Regulatory and health technology assessment bodies have increasingly considered external or synthetic controls to augment or substitute for concurrent controls, particularly in rare diseases and rapidly evolving therapeutic areas (e.g., oncology) where recruitment is challenging and the standard of care changes over time [5]. As outlined in recent primers, the validity of synthetic control comparisons depends on careful harmonization of different populations, outcome definitions, and covariates; appropriate statistical adjustment (e.g., propensity scores, Bayesian networks); and robust sensitivity analyses to assess bias and generalizability [5]. When such conditions are met, synthetic controls have supported label expansions and informed comparative effectiveness; however, their credibility rests on the quality and comparability of the underlying data sources and the methods used to align them [2,3,5]. Accordingly, references to synthetic or external controls are included here as broader background only and should not be interpreted as a direct application evaluated in the present study. Likewise, any relevance to regulated development environments should be understood in an operational sense only; the present study does not provide formal mapping to CDISC, GCP, or regulatory acceptance criteria.

In parallel, advances in generative modeling have accelerated the use of synthetic data to expand access to sensitive information [1]. In oncology and other domains, synthetic data has been used for data augmentation, detection and prognosis tasks, and exploratory analyses when real data are unavailable or sensitive [6]. Generative adversarial networks and related deep generative approaches have produced high-fidelity images and tabular datasets, but they also present challenges, including vulnerability to privacy attacks, variable fidelity and diversity across subgroups, and the need for rigorous quality assessment to avoid amplifying bias or generating implausible outliers [1,4,6,7,8]. Medical synthetic data should therefore be evaluated in terms of fidelity, diversity, and generalization, with privacy assessment and safeguards selected for the intended release context [1,3,6].

Evidence also suggests that, when properly generated and evaluated, synthetic clinical datasets can serve as effective proxies for real data in secondary analyses. Replication studies have demonstrated a high concordance between results derived from synthetic variants and those from original trial datasets across univariate, bivariate, and multivariate analyses, including survival models, with overlapping confidence intervals and consistent inferential conclusions [2,9]. Such findings support the use of synthetic datasets for hypothesis generation, benchmarking methods, and preliminary decision support, with the option to employ verification servers or dynamic borrowing for confirmatory analyses when required by regulators or sponsors [2,3].

Against this background, an important distinction is required between schema conformance and statistical fidelity. Schema conformance refers here to adherence to the study-specific ALS/eCRF structure, including forms, fields, permitted values, visit scheduling, and associated operational rules. Statistical fidelity refers to reproduction of selected empirical distributions and dependency patterns observed in a matched reference study. Many synthetic-data approaches primarily optimize statistical or task-based fidelity, whereas operational study reconstruction may require additional study-specific conditioning or postprocessing. The contribution evaluated in this study is not a new general-purpose generative model; it is an integration of ALS/eCRF-native study reconstruction and visit scheduling, empirically informed module-level synthesis, and configurable, reproducible fault injection for validation-oriented workflows before real data are available. The evaluation does not establish superiority over GAN-based, VAE-based, copula-based, or other synthesis approaches, and references to regulated clinical development describe the operational context rather than formal CDISC or GCP mapping or regulatory qualification [3,6].

The aim of this study was to develop and evaluate a modular framework for generating ALS/eCRF-conformant synthetic clinical trial datasets by combining ALS-defined study structure with selected empirical patterns learned from a matched reference study. For operational and validation workflows before real trial data are available, the framework prioritizes three objectives: (i) conformance to the target eCRF schema and ALS-defined scheduling; (ii) reproduction of selected distributions and relationships for categorical variables, continuous measures, dates, and missingness; and (iii) transparency and reproducibility through explicit scenario construction, controlled insertion of operational deviations, and deterministic per-patient seed assignment.

## 2. Materials and Methods

### 2.1. Study Design

SYNDATA is a modular framework for generating synthetic clinical trial data for operational validation, including the development and testing of reports, data listings, dry runs, edit checks, and centralized or risk-based monitoring dashboards. It is intended particularly for study phases in which the target eCRF has been defined but sufficient study data are not yet available. The approach uses two inputs: (1) study metadata defined in the Architect Loader Spreadsheet (ALS) and (2) selected empirical distributions learned from a matched reference study. The workflow comprises preprocessing, generation, postprocessing, and controlled insertion of operational deviations. Patient-specific timeline synthesis is parallelized, and each patient is assigned an independent seeded pseudorandom-number generator. The design prioritizes modularity, reproducibility, and explicit control over ALS-defined structure and scenarios. GAN- and VAE-based models were not evaluated as comparators because the study did not prespecify a comparative benchmark with harmonized structural constraints, preprocessing, tuning, repeated runs, and common evaluation criteria. No claim of comparative superiority is made.

### 2.2. Conceptual Positioning Relative to Other Synthesis Approaches

SYNDATA was developed with a primary objective that differs from that of many general-purpose synthetic tabular-data generators (Table 1). Deep generative models, including GAN- and VAE-based approaches, can model complex multivariable distributions, whereas conventional statistical synthesis methods can reproduce prespecified marginal or conditional structures using transparent statistical models. SYNDATA instead prioritizes explicit reconstruction of the target ALS/eCRF structure, visit-schedule logic, deterministic generation, and configurable fault injection for operational validation. These are differences in design emphasis, not evidence of comparative performance. A rigorous empirical comparison would require prespecified comparators, harmonized structural constraints and preprocessing, method-specific tuning, repeated runs, and common evaluation criteria.

### 2.3. Data Sources and Inputs

ALS (Architect Loader Spreadsheet): The ALS defines the eCRF schema, including forms or modules, fields, data types, permitted values, folders, and events such as Screening and Visits. It specifies scheduling through TargetDays and OverDueDays, and identifies fields requiring coding against external dictionaries, including MedDRA and WHODrug. In this implementation, the target modules were based on operational structures used in clinical development, including modules derived from the AstraZeneca Clinical Data Standards library.

Reference study data: These data provide selected empirical distributions, categorical dependencies, date-difference densities, and patterns of conditional missingness. ALS definitions take precedence when discrepancies occur, and reference-study rows that violate ALS enumerations are removed.

External dictionaries and configuration: Coding dictionaries (e.g., MedDRA, WHODrug) and user-provided configuration files are used for counters, metadata handling, and Subject Matter Expert (SME) rules that guide scenario construction and postprocessing.

### 2.4. Preprocessing

ALS parsing and typing: The ALS was parsed to identify event types, such as Screening, Rescreening, and Visit, as well as form-level schemas and field properties. Fields were classified as categorical, free text, single-value constants, time, date/datetime, float, or medical examination result. Scheduling constraints were extracted from TargetDays and OverDueDays for each folder. Fields requiring external dictionary coding were identified from the ALS.

Reference-data cleaning and reconciliation: Rows containing categorical pairings flagged by the prespecified cleaning procedure as potentially erroneous or highly specific were removed before model fitting. Rows with values that violated ALS enumerations were also removed. Naming inconsistencies among modules, columns, and values in the reference data and ALS were reconciled where feasible. When an ALS-specified module, field, or value was absent from the reference data, it was generated according to ALS rules. The effects of the categorical-pairing exclusion on statistical fidelity and disclosure risk were not evaluated separately.

### 2.5. Categorical Modeling and Density Estimation

A minimal subset of categorical predictors was selected to generate other categorical variables through direct mappings. For selected variables, module-specific Bayesian networks were trained on cleaned reference-study data using pgmpy, hill-climb score-based structure learning with the default BIC-d score, a maximum in-degree of 2, and no required or forbidden edges. The fitted networks were then used for joint sampling [10]. Numeric variables were sampled uniformly within ALS-defined ranges or from kernel density estimates (KDEs) fitted to reference data. For the continuous ECG result variable EGORRES, the KDE bandwidth was selected using Silverman’s rule, providing a data-adaptive smoothing parameter based on the characteristics of the available observations. Other KDE-based components were not regenerated or reoptimized for this revision. No variable-specific cross-validation or sensitivity analysis was performed; the revised EGORRES analysis therefore represents a reproducible rule-based update rather than evidence of universally optimal bandwidth selection. Applicable ALS-defined constraints were checked after sampling. The evaluation did not separately quantify tail calibration, rejection, or clipping behavior. Missingness patterns were reapplied after generation through conditional rules [11].

### 2.6. Patient Scenario Creation

**Figure 1:**
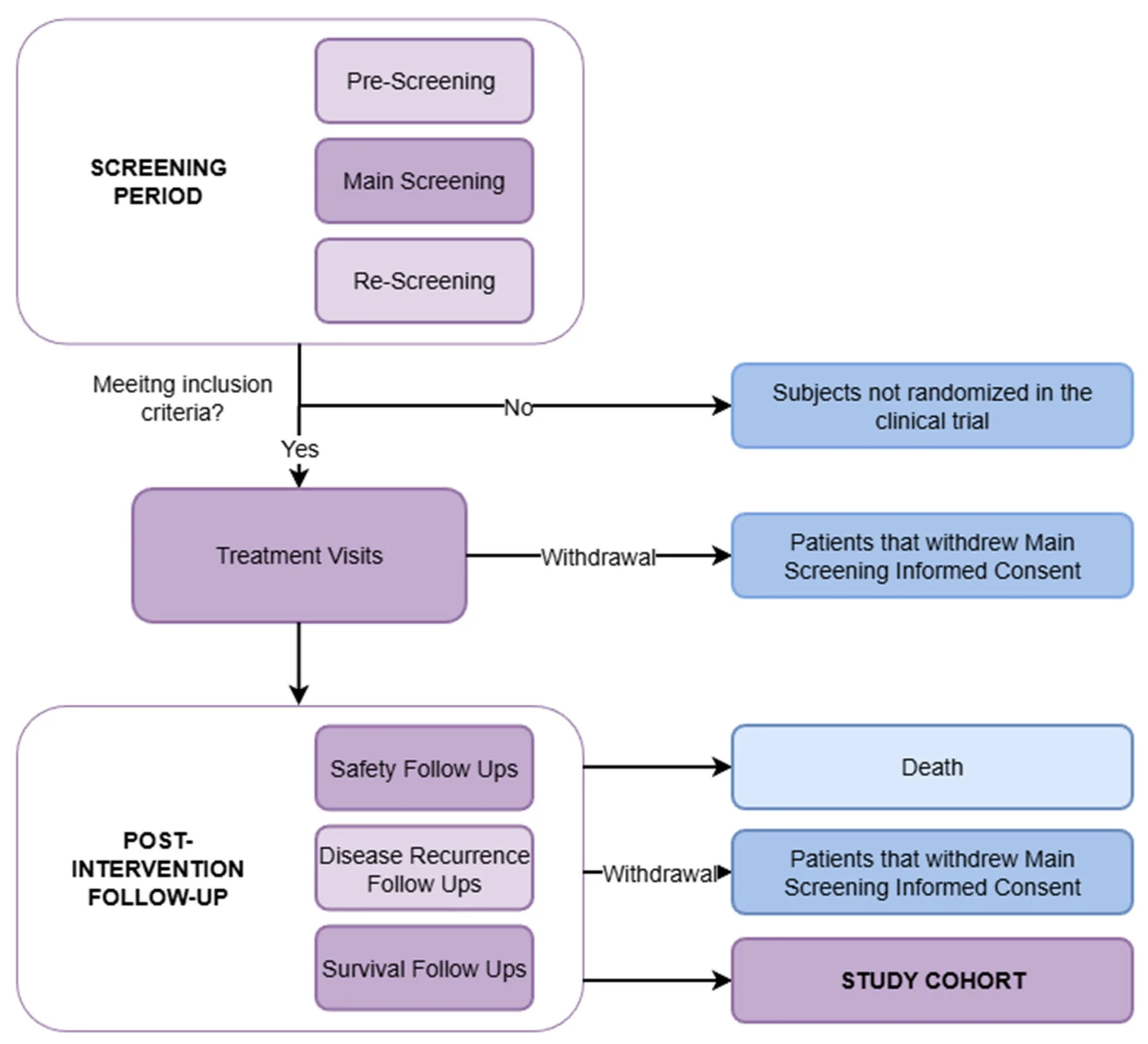
Example of a patient scenario and its corresponding modules during synthetic-data generation for an adjuvant oncology trial. The ALS defines the mapping of visits and folders.

A patient-specific timeline was constructed from the ALS folder schedules shown in Figure 1. Scheduled events were placed at patient target days within TargetDays ± OverDueDays. The number of failed screenings, early-termination status, and reasons for end of participation were determined stochastically and standardized to coded representations. Unscheduled events, including adverse events (AE) and concomitant medications (CM), were inserted, and selected temporal-overlap scenarios (AE-AE, AE-CM, CM-CM, and MH-MH) could be generated. An AE-CM temporal overlap represents concurrent records only and does not establish a clinically meaningful link between a specific adverse event and a medication prescribed for its management. For each serious adverse event (SERAE), a corresponding AE was ensured and linked through folder_nr and an auxiliary flag. If a patient had no AE or CM, an explicit no-event row was added to the relevant module.

Operational deviations generated by the Noise Tool included rescreening_out_of_window, visit_out_of_window, additional_rescreening, two_visits_same_day, and missing_visit, as defined by ALS timing windows and sequence logic. Each patient received strategy tags, such as Screen Failure or Male, that guided deterministic behavior in subsequent steps.

### 2.7. Strategy-to-Data Translation

Each patient was assigned an object that translated predefined strategies into deterministic data actions, including value assignments specified as module-column-value triplets and the suppression of modules that were incompatible with a strategy.

### 2.8. Synthetic Data Generation

Event-level generation was performed by the module using a standardized procedure. Row structures were initialized with folder-specific identifiers and algorithm metadata, and modules describing the same event shared common values unless ALS constraints or a “CAT” suffix required preventing propagation. Categorical variables were sampled from trained Bayesian networks and learned mappings, with controlled injection of ALS-valid values absent from the source data to ensure enumeration coverage. Free-text fields were populated with “Synthetic <column_name>” or left missing. Temporal variables were generated using uniformly sampled times, event-aligned initial dates, and empirical date-difference distributions for subsequent dates. Numeric variables were sampled uniformly within ALS-defined ranges or from KDEs trained on the base data; analytes with ALS ranges were generated from normal distributions with most values within range, whereas analytes without empirical data were generated from N(0, 1) [12]. For ALS-required fields absent from the base data, predefined defaults were used: uniform sampling across categorical enumerations, event dates, uniform times, uniform values on [0.0, 1.0) for unrestricted numeric fields, synthetic strings for free text, and N(0, 1) for analytes. Cross-module relationships were not learned systematically from the source data; most variables followed module- and type-specific rules, while selected within-module categorical dependencies were modeled using Bayesian networks. A limited set of prespecified cross-module rules supported operational consistency, including serious adverse event–adverse event linkage and selected patient-status, pregnancy-related, dosing, and temporal dependencies; these rules do not constitute comprehensive ontology- or knowledge-guided modeling of clinical relationships across domains.

### 2.9. Binary Mask for Conditional Missingness and Rule Enforcement

Binary masks were used to apply selected conditional missingness patterns and rule-based dependencies observed in the reference study. During preprocessing, recurring patterns were identified, including cases in which the observed value of one field implied that another field should remain empty or should not be collected. After initial value generation, masks were applied at the record level to nullify fields according to these observed conditions and protocol logic [13]. This procedure reproduces selected observed conditional missingness patterns but does not identify or formally simulate missing completely at random, missing at random, or not missing at random mechanisms. No selection model, pattern-mixture model, or sensitivity analysis for informative missingness was implemented.

### 2.10. Postprocessing

Postprocessing enforced ALS and user requirements by applying temporal constraints, conditional missingness masks, required metadata, synchronization of within- and cross-module counters, and formatting of numeric and partial-date fields. Dictionary-coded variables were populated with externally supplied MedDRA 27.1 and WHODrug Global B3 (1 September 2024) mappings. Fixed dictionary versions were maintained across runs. SYNDATA does not provide independent terminology governance; synonym resolution, dictionary-version migration, and cross-release harmonization remain functions of the external coding workflow and were not evaluated.

### 2.11. Validation and Analytical Methods

Jensen–Shannon distance and Cramér’s V were reported as continuous descriptive measures [14,15]. Lower values were interpreted as indicating closer agreement between the compared distributions. No externally validated or study-calibrated acceptance thresholds were available; therefore, the metrics were not categorized into qualitative similarity bands and were not interpreted as evidence of formal equivalence or universal acceptability. For each categorical variable, Cramér’s V was calculated from a contingency table crossing dataset origin (source versus synthetic) with observed category levels. In this formulation, lower values indicate a weaker association between dataset origin and category composition. Validation also assessed schema conformity, temporal coherence, categorical coverage, selected dependency structures, and conditional missingness. The reference study served as an empirical benchmark rather than as a control group for causal or comparative inference. Numeric 95% bootstrap confidence intervals for Jensen–Shannon distance and Cramér’s V were reported in Table 2. Because records may be clustered within patients, the bootstrap procedure was performed using patient-level resampling rather than independent record-level resampling. The confidence intervals quantify the uncertainty associated with the reported descriptive similarity measures.

### 2.12. Tools and Software

The framework was implemented in Python version 3.12.3. The workflow includes a custom ALS parser and scenario engine, Bayesian networks trained on cleaned reference data for selected categorical variables, KDEs for numeric variables and date differences, and per-patient NumPy random generators for deterministic parallel execution. Configuration is managed through coded fields and externally supplied MedDRA and WHODrug mappings [16]. In the evaluated computing environment, generation of the 373-patient cohort using 16 CPU cores required 55 min 56 s of wall-clock time. Reported CPU time was 7 h 29 min 35 s, corresponding to 50.24% CPU efficiency relative to available core-wall time. Reported memory use was 37.24 GB of 256 GB. These values constitute an environment-specific implementation benchmark rather than a formal scalability analysis.

Because the study evaluated synthetic-data properties rather than clinical hypotheses, the analyses focused on descriptive statistics, distributional discrepancy metrics, and rule-based consistency checks. Statistical significance testing was not used because the study did not specify inferential estimands or equivalence margins and because record-level observations were clustered within patients. The reported metrics describe observed discrepancies and do not establish formal equivalence. The source and synthetic cohorts each included 373 patients. No formal power analysis was conducted because the study was not designed as a hypothesis-testing experiment.

### 2.13. Noise Tool

**Figure 2:**
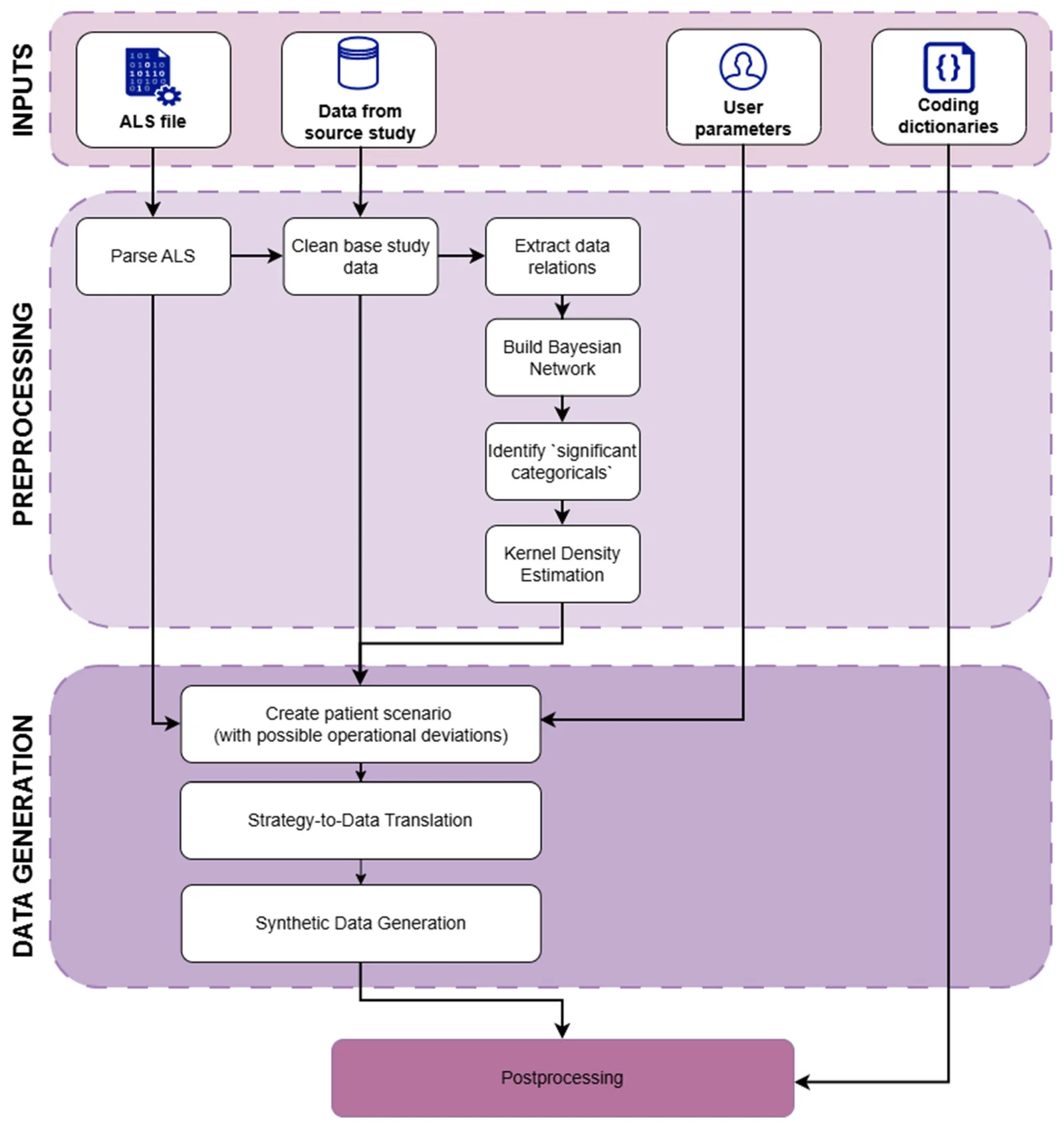
SYNDATA synthetic clinical data-generation workflow. The diagram summarizes ALS and reference-study input processing, schema reconciliation, patient-scenario creation, module-specific record generation, postprocessing, controlled insertion of noise scenarios, and validation-oriented output preparation.

Scenario-related deviations were introduced during scenario construction, whereas additional user-defined errors were added after generation to isolate their effects from the core synthesis. During preprocessing, invalid categorical pairings were removed and ALS specifications were prioritized when conflicts occurred. ALS-specified modules or fields that were absent from the reference data were generated to ensure structural completeness. Patient-level scenario assignment was parallelized with separate seeded NumPy random generators and fitted Bayesian-network and KDE components were serialized and reused to support deterministic execution. The overall SYNDATA generation workflow is summarized in Figure 2.

The Noise Tool introduces controlled, reproducible defects into otherwise clean synthetic datasets to test validation rules and reporting-pipeline robustness. Its 23 configurable scenarios include format and range violations, timeline inconsistencies, and structural or cross-dataset conflicts (Supplementary Table S1). Each perturbation has a configurable frequency, magnitude, and target location, allowing reproducible testing of both simple checks and cross-table constraints.

## 3. Results

### 3.1. Overview of Synthetic Clean Datasets and Controlled Error Scenarios

SYNDATA generates clinical datasets aligned with a target study design before sufficient real study data are available. The framework uses the target ALS and reference data from a matched study. For each synthetic patient, it constructs an event timeline from the ALS visit matrix and populates modules using a custom variable taxonomy and dedicated generation methods. Although a broad set of modules was generated, the main text reports a representative subset selected to span key clinical and operational domains, to include different categorical structures, and to illustrate both close descriptive agreement and known limitations.

Because the central use of synthetic data in this framework is the testing of databases and programming tools, the objective is to generate feasible value combinations under the target design rather than to reproduce every empirical distribution exactly. Patient-specific scenarios can influence record burden and selected variables; synthetic distributions are therefore not expected to match the reference data uniformly.

To support validation of quality-control rules, SYNDATA provides 23 configurable scenarios that can be injected into otherwise clean synthetic data. Scenarios include both feasible value combinations and deliberate simulations of common data-entry or operational errors. A single scenario may affect one or multiple datasets. Users select scenarios and, for each, specify the intensity (percentage of affected rows or patients) and, where applicable, the targeted dataset(s).

In the present analysis, we report results for five representative scenarios spanning different data types and validation classes: Date Threshold—Different Datasets, Visit Outside of Window, Result Range Threshold, Incorrect Randomization, and a structural conflict (Duplicated Lines or Two Visits Same Day). Both the source and synthetic cohorts included 373 patients.

Synthetic and source data were compared descriptively using Jensen–Shannon distance, Cramér’s V [14,15], distribution plots, and rule-based validation measures appropriate to the variable type and operational objective. Both metrics are continuous measures of distributional discrepancy, with lower values indicating closer agreement; no universal equivalence or acceptability threshold was applied. Record-level comparisons characterize category composition among available records and do not establish similarity in event incidence, records per patient, the proportion of patients with an event, or overall patient-level record burden. Absolute record-count differences should therefore be interpreted separately from within-record category distributions (Table 2).

Figure 3, Figure 4, Figure 5, Figure 6 and Figure 7 present selected distributional examples, and Figure 8, Figure 9 and Figure 10 illustrate dependency structures and scenario diversity. Figure 11, Figure 12, Figure 13, Figure 14, Figure 15 and Figure 16 show examples of deliberately introduced errors, whereas Figure 16 shows temporal-overlap scenarios that are not inherently erroneous.

Jensen–Shannon distance and Cramér’s V are reported as continuous descriptive measures; a value of 0 denotes identical observed category distributions. Statistical significance testing was not used because no inferential estimands or equivalence margins were prespecified and record-level observations were clustered within patients.

**Figure 3:**
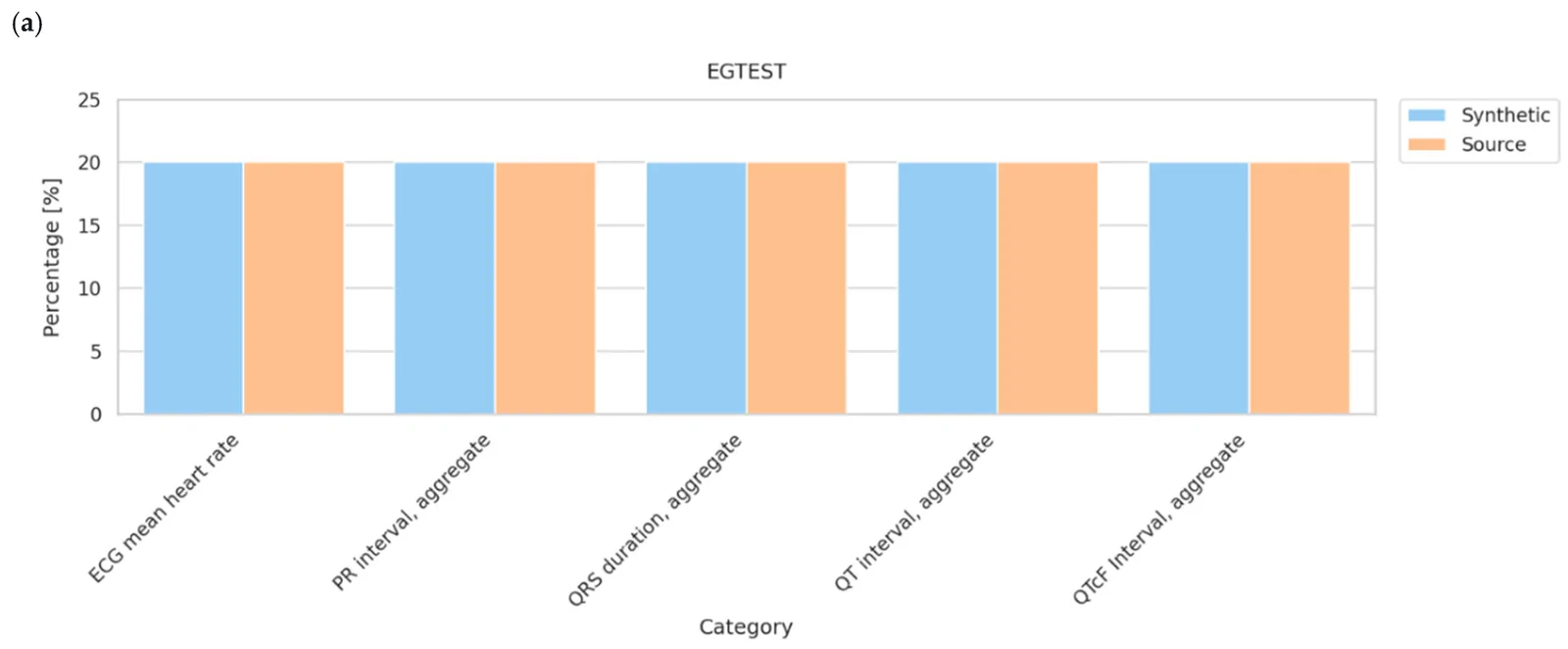

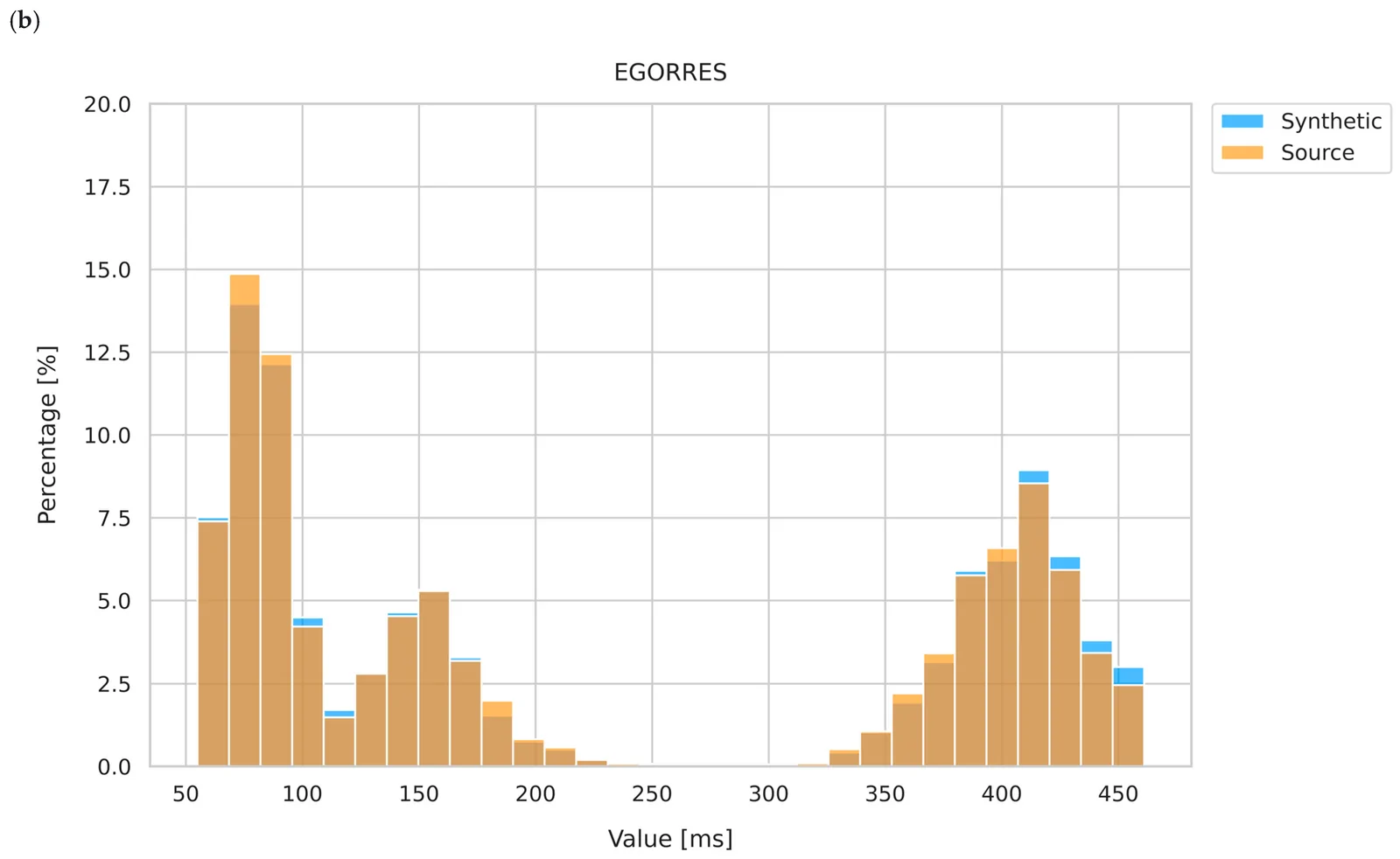
Comparison of ECG-related variables in the synthetic and source datasets. (**a**) EGTEST (ECG test name), shown as category proportions; (**b**) EGORRES (ECG original result), shown as a continuous distribution in milliseconds. Table 2 reports categorical metrics for EGTEST only and does not provide quantitative validation of the continuous EGORRES distribution.

Figure 3 illustrates two complementary aspects of the EG domain. Panel A compares the categorical distribution of ECG test names, whereas Panel B presents continuous ECG results in milliseconds. The displayed EGORRES distributions show close descriptive agreement in their principal concentration regions; however, no formal equivalence test or tail-calibration analysis was performed.

**Figure 4:**
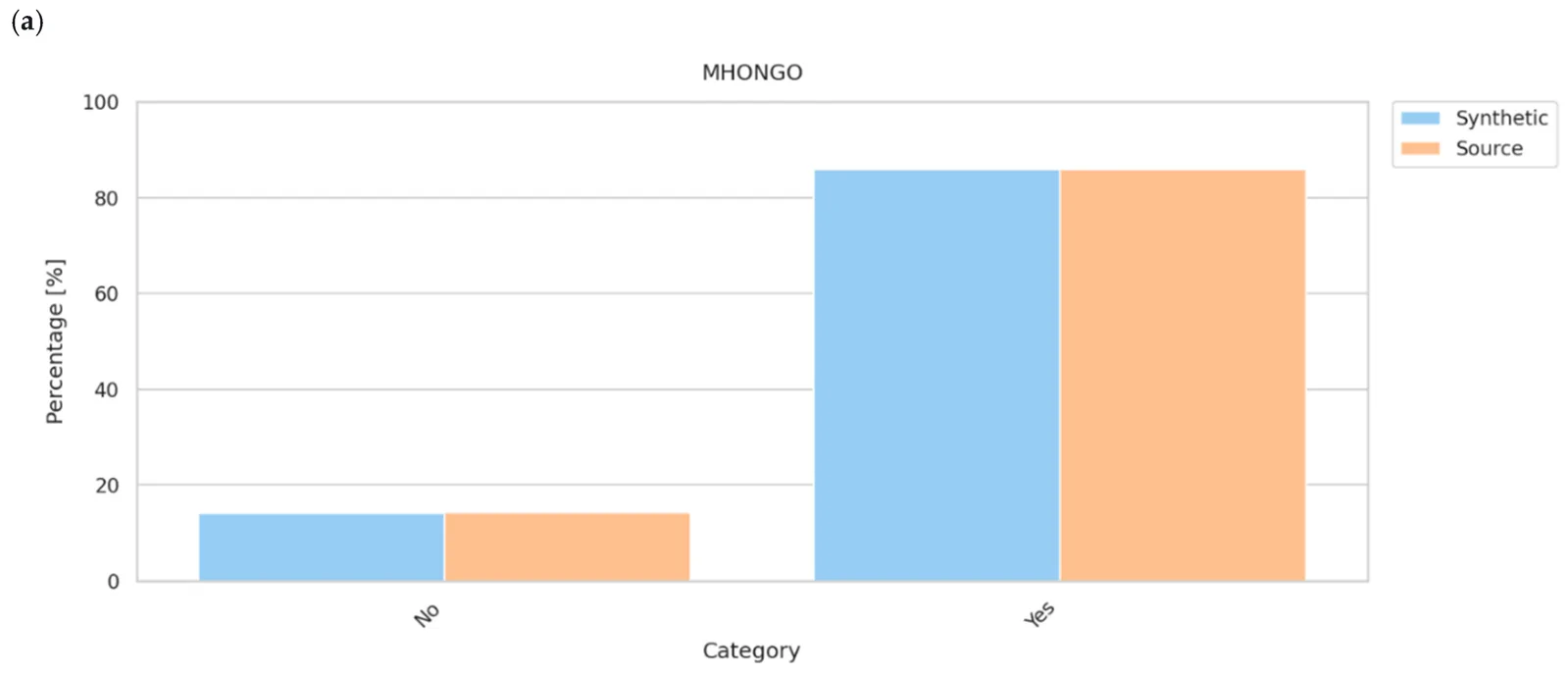

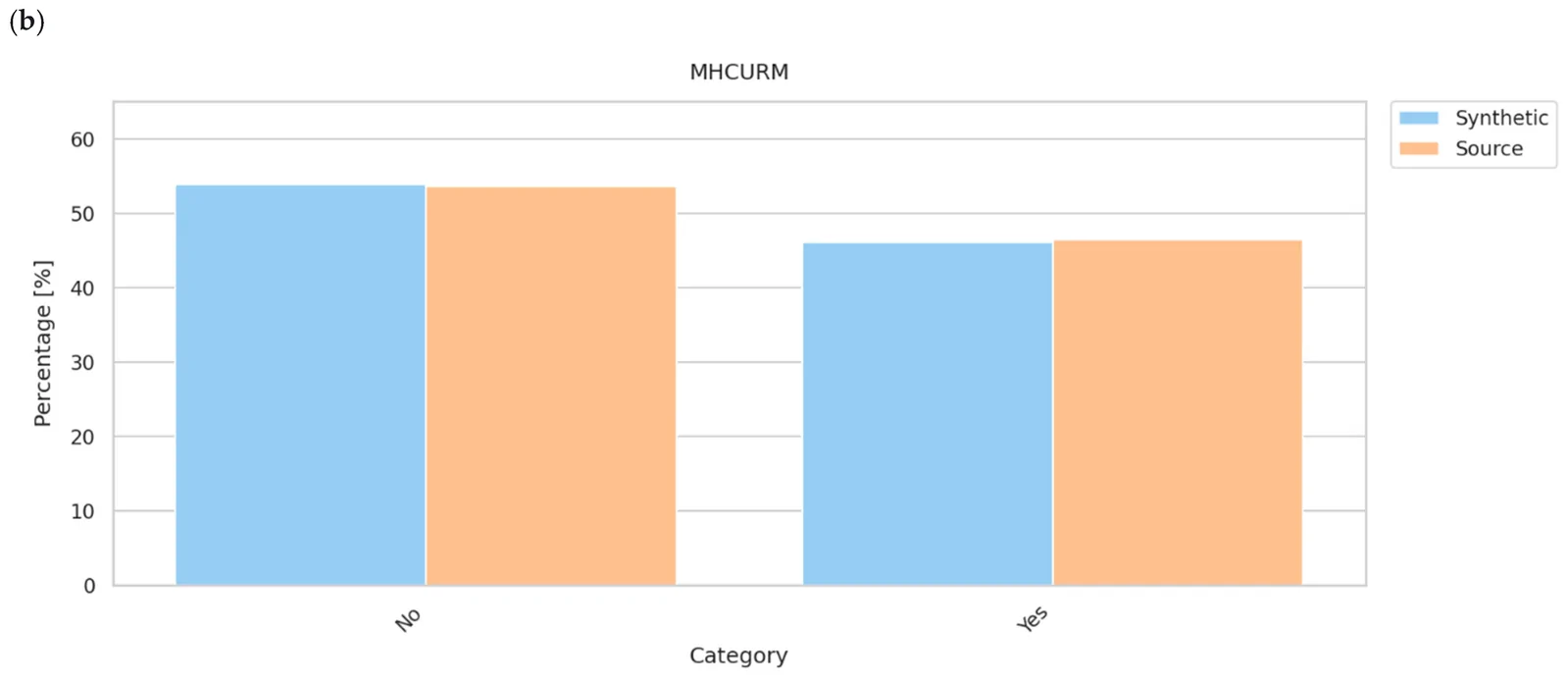
Comparison of categorical distributions in the MH module: (**a**) MHONGO and (**b**) MHCURM. The bar plots show close descriptive agreement for the displayed binary medical history fields; see Table 2.

Figure 4 shows a close descriptive agreement for the displayed MHONGO and MHCURM category proportions. This result applies to these selected record-level fields and should not be generalized to the full medical-history domain.

**Figure 5:**
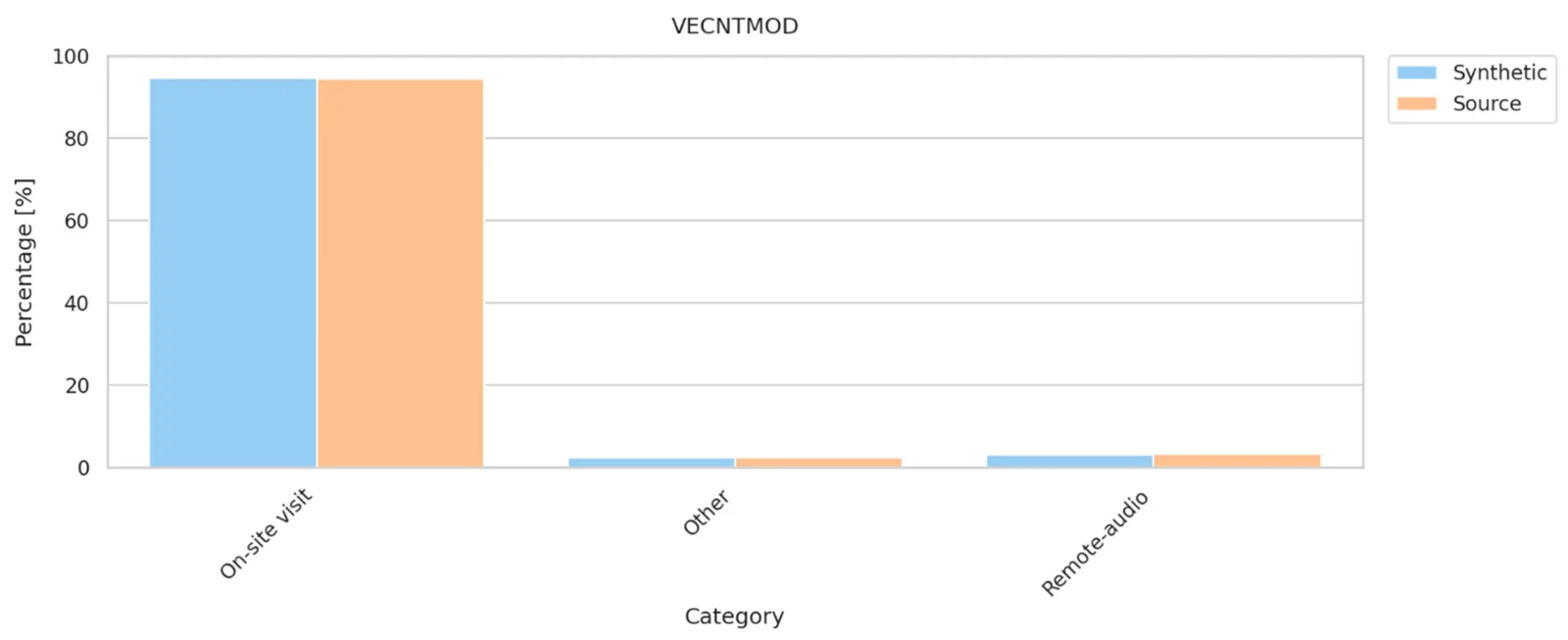
Comparison of the VECNTMOD categorical distribution in the VISIT module. The displayed record-level proportions show a small observed discrepancy; see Table 2.

Figure 5 presents the VECNTMOD contact mode distribution. The displayed proportions show close descriptive agreement within the evaluated source-synthetic comparison.

**Figure 6:**
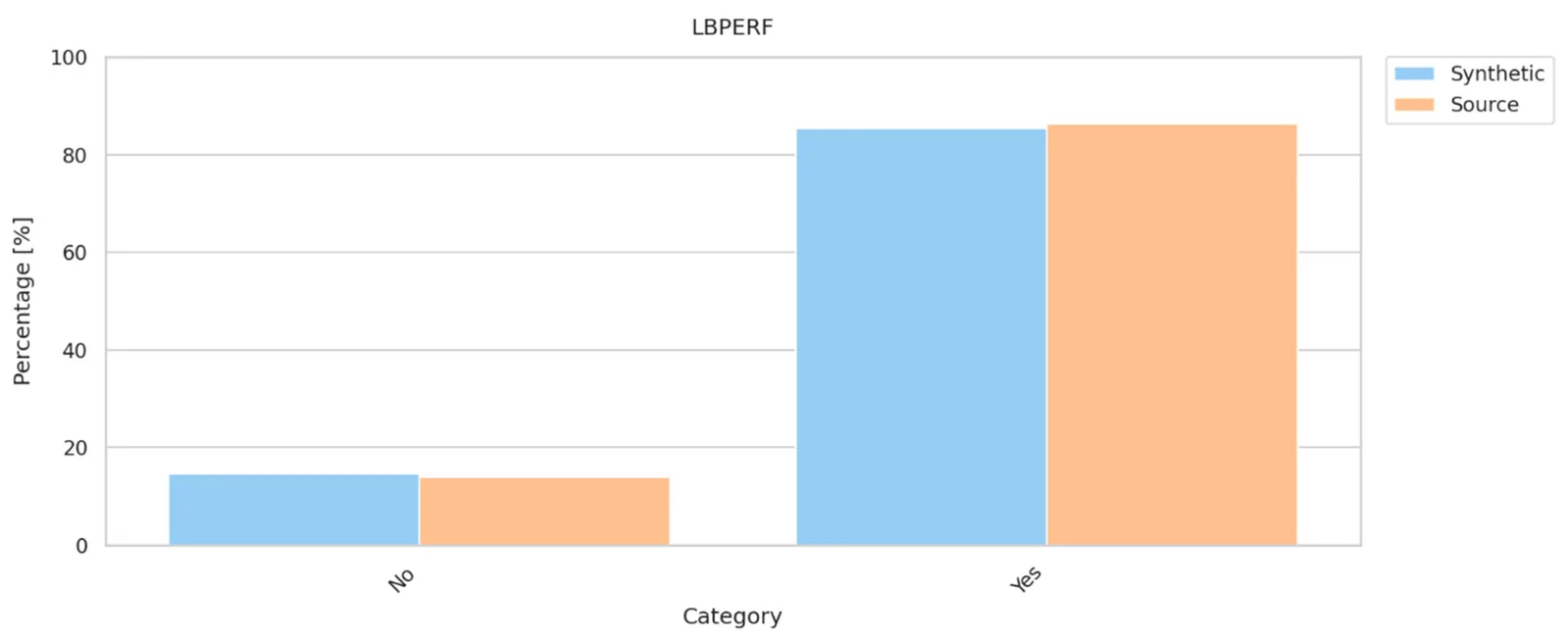
Comparison of the LBPERF categorical distribution in the laboratory module. The displayed record-level proportions show close descriptive agreement; see Table 2.

Figure 6 compares the categorical LBPERF workflow fields. It does not evaluate continuous laboratory analytes.

**Figure 7:**
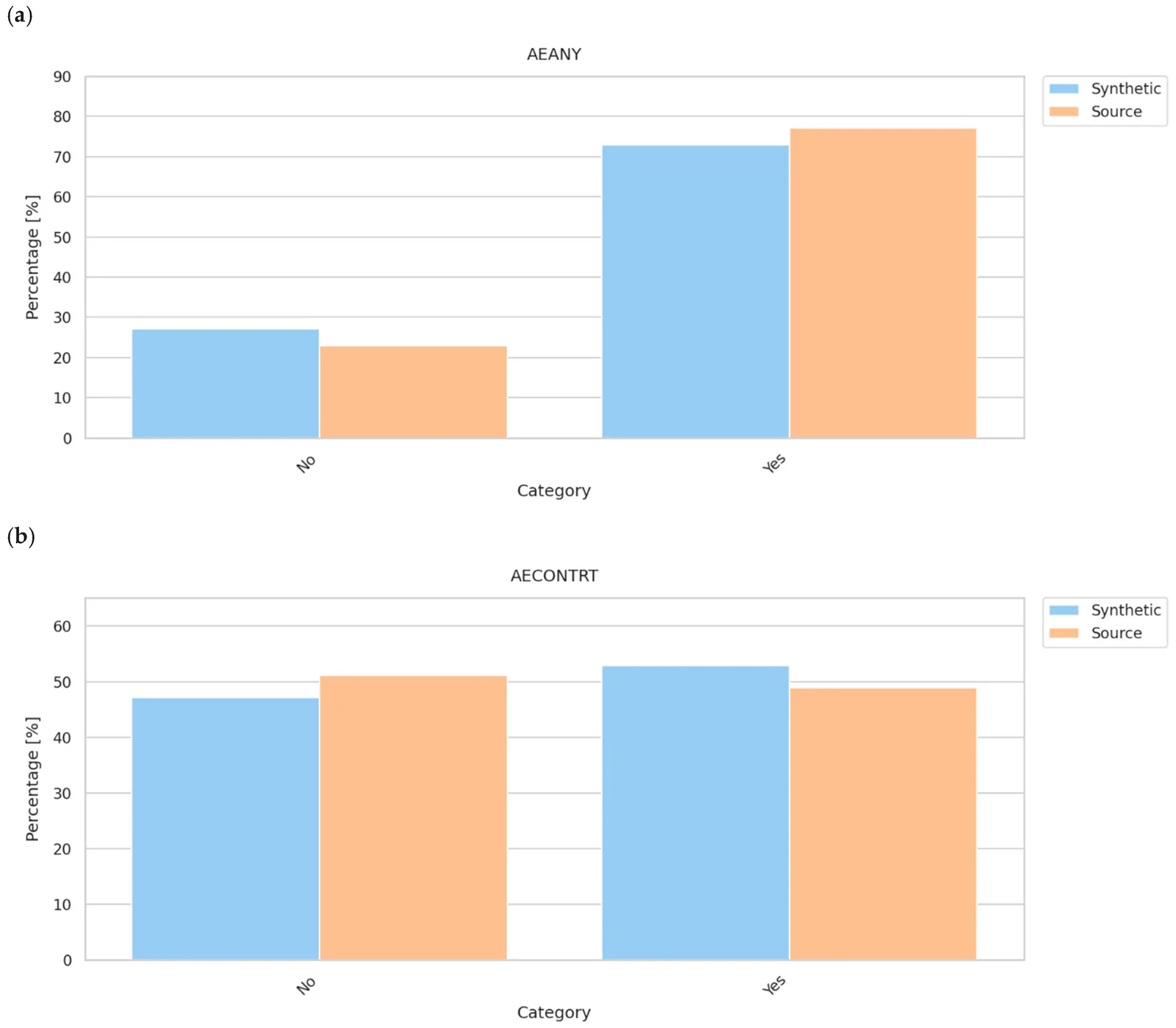
Comparison of categorical distributions in the AE module: (**a**) AEANY and (**b**) AECONTRT. The bar plots show category proportions in the synthetic and source datasets; see Table 2.

Figure 7 shows small observed discrepancies in the record-level category composition of AEANY and AECONTRT. The large differences in absolute record counts mean that these comparisons do not establish similarity in the patient-level adverse event burden.

**Figure 8:**
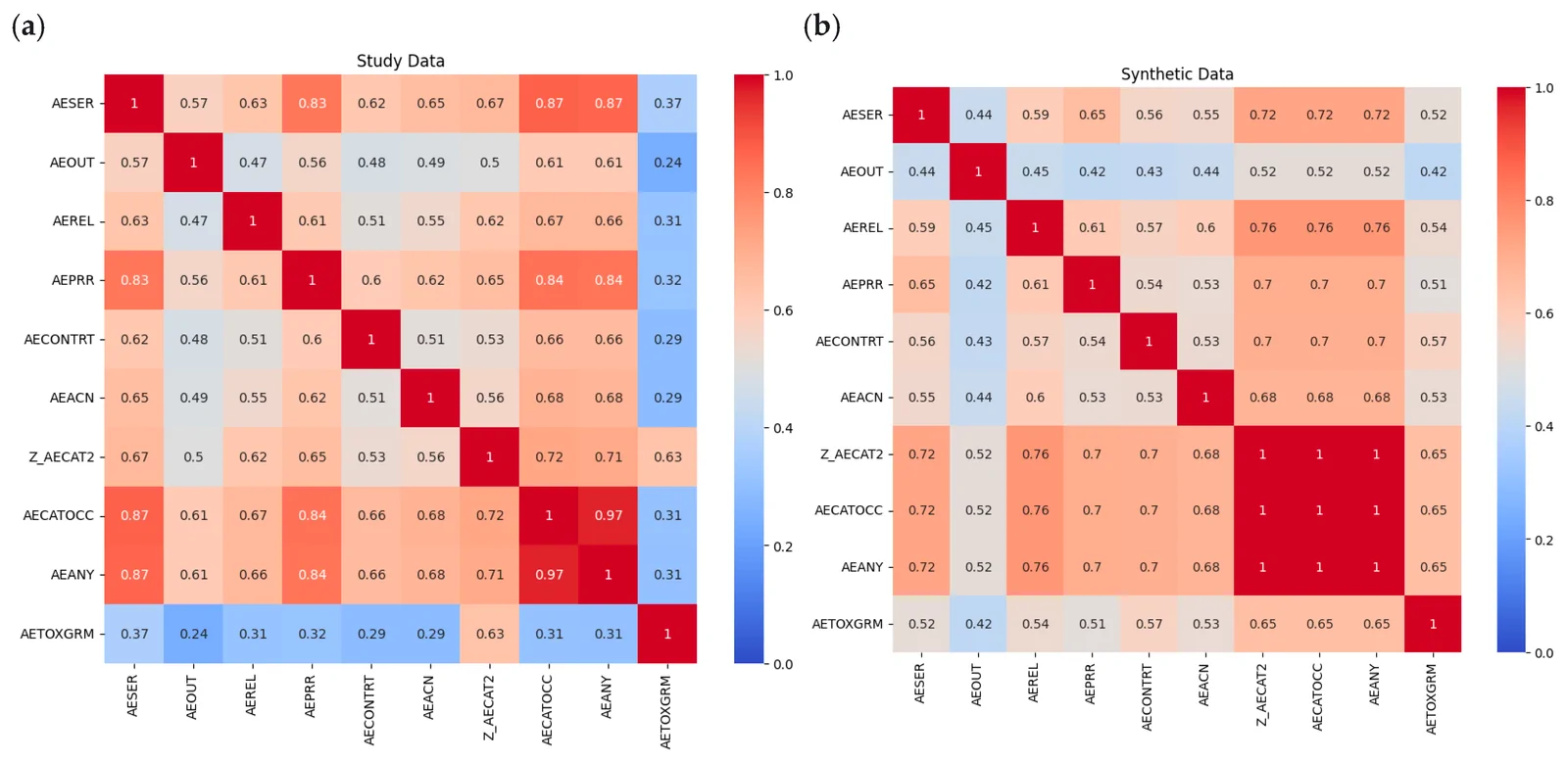
Pairwise normalized mutual information (NMI) matrices for ten selected categorical AE variables in the source and synthetic datasets: (**a**) source dataset; (**b**) synthetic dataset. The same matrices were used for the quantitative matrix comparison. Color intensity indicates NMI strength; the display should not be interpreted as a Pearson correlation.

For the ten selected AE variables in Figure 8, the difference between the source and synthetic NMI matrices had a Frobenius norm of 1.3887. The relative Frobenius norm, defined as ||NMI_source–NMI_synthetic||F/||NMI_source||F, was 0.2108. The mean absolute NMI difference was 0.1153. These summaries were calculated across all 100 elements of the 10 × 10 matrices, including the diagonal and symmetric duplicates; the matrix contains 45 unique off-diagonal variable pairs. The results show measurable differences and should not be interpreted as identical preservation of the selected dependency structure. They apply only to the displayed AE variables and do not establish multivariable fidelity across all modules. Uncertainty across independently generated cohorts was not assessed.

**Figure 9:**
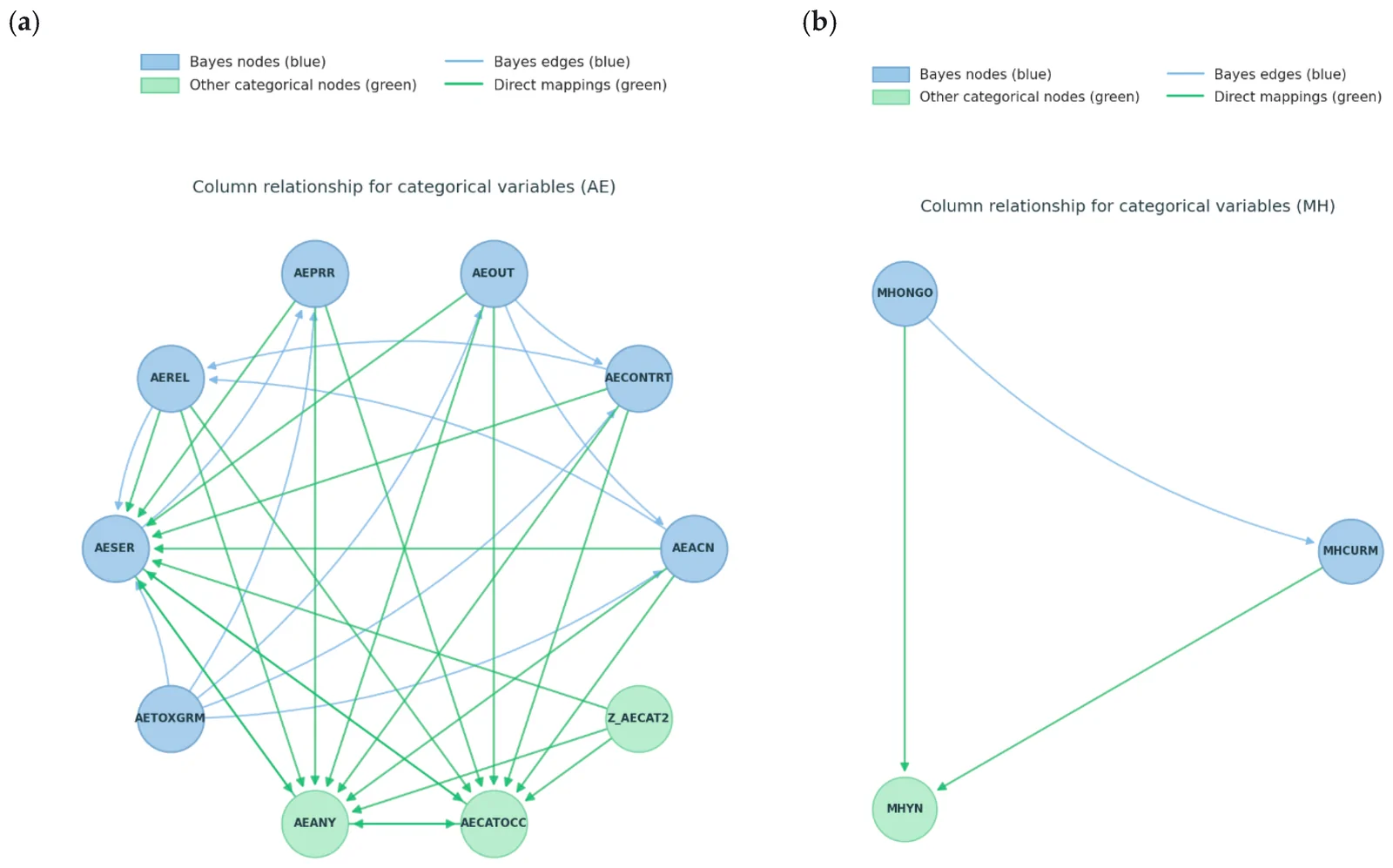
Learned Bayesian-network structures for selected categorical variables in the (**a**) AE and (**b**) MH domains. Blue nodes and edges represent variables included in the fitted Bayesian networks; green nodes and edges represent other categorical variables and direct mappings. Edge directions describe the fitted generative factorization and should not be interpreted as causal.

Figure 9 illustrates Bayesian-network structures learned from the reference study and used during synthesis in the AE and MH domains. The models reproduce selected empirically observed within-module categorical dependencies, rather than sampling each variable independently. The graphs are representative examples; they do not demonstrate clinical plausibility, causal relationships, or fidelity across all domains.

**Figure 10:**
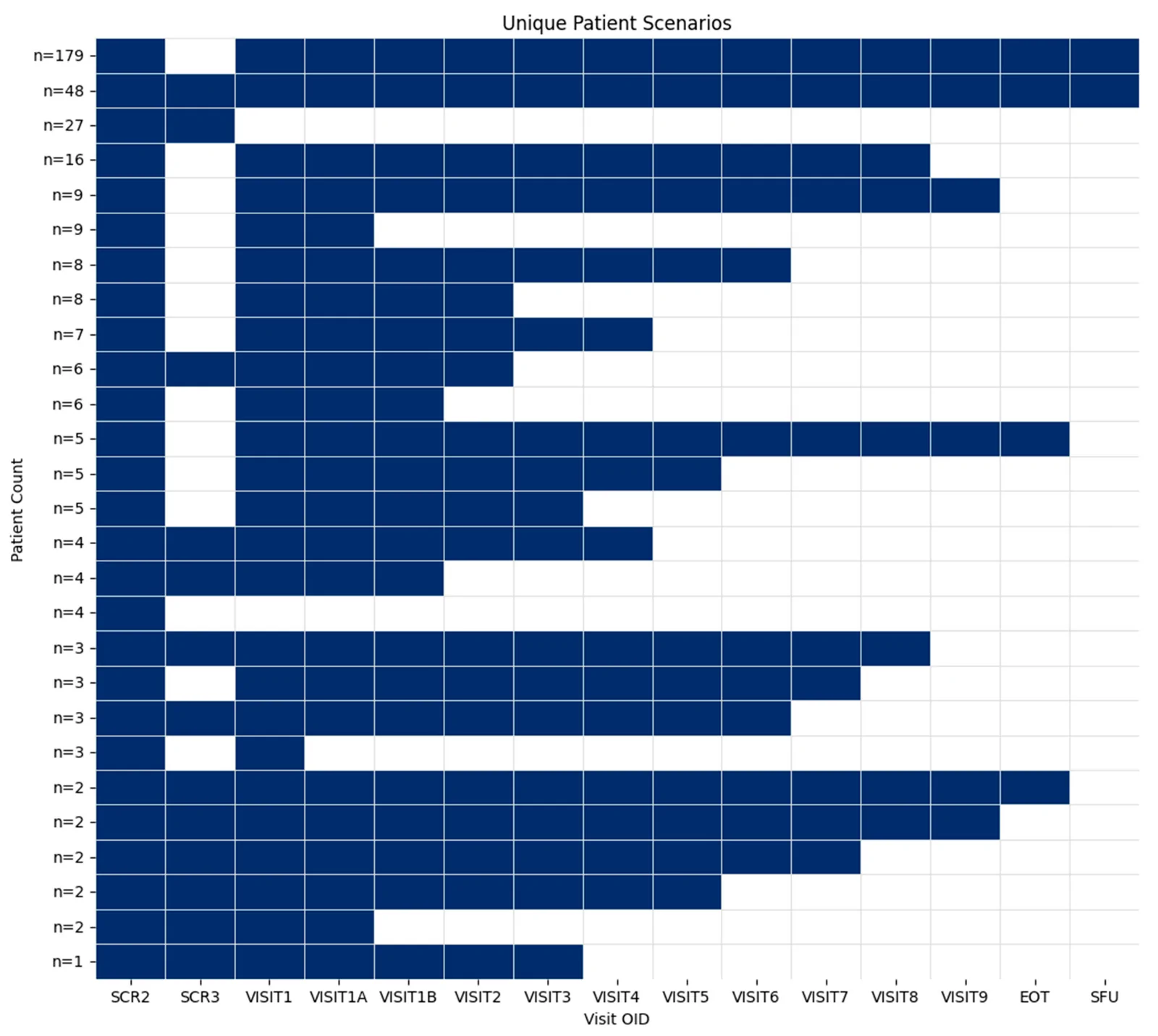
Unique patient scenarios illustrate protocol-consistent patient trajectories within the synthetic cohort.

Figure 10 summarizes protocol-consistent operational trajectories in the synthetic cohort, including study completion, screening failure, and early termination. The scenario-based design supports testing across multiple operational pathways; it is not a calibrated model of disease progression or of treatment response.

### 3.2. Noise-Induced Errors

**Figure 11:**
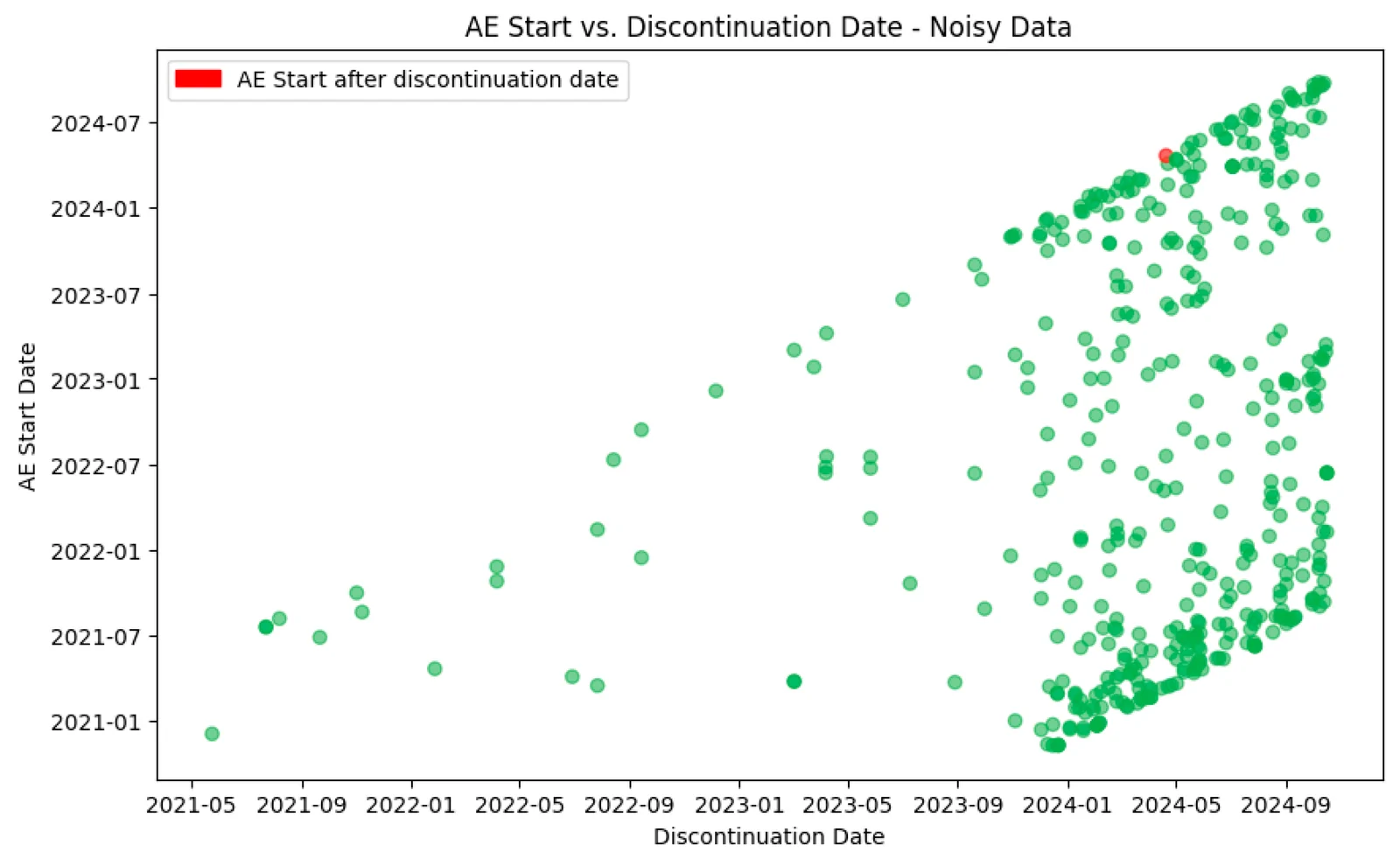
Example of a cross-dataset date inconsistency in which an AE start date occurs after discontinuation has taken place. The modification is applied with user-defined intensity and a fixed random seed.

**Figure 12:**
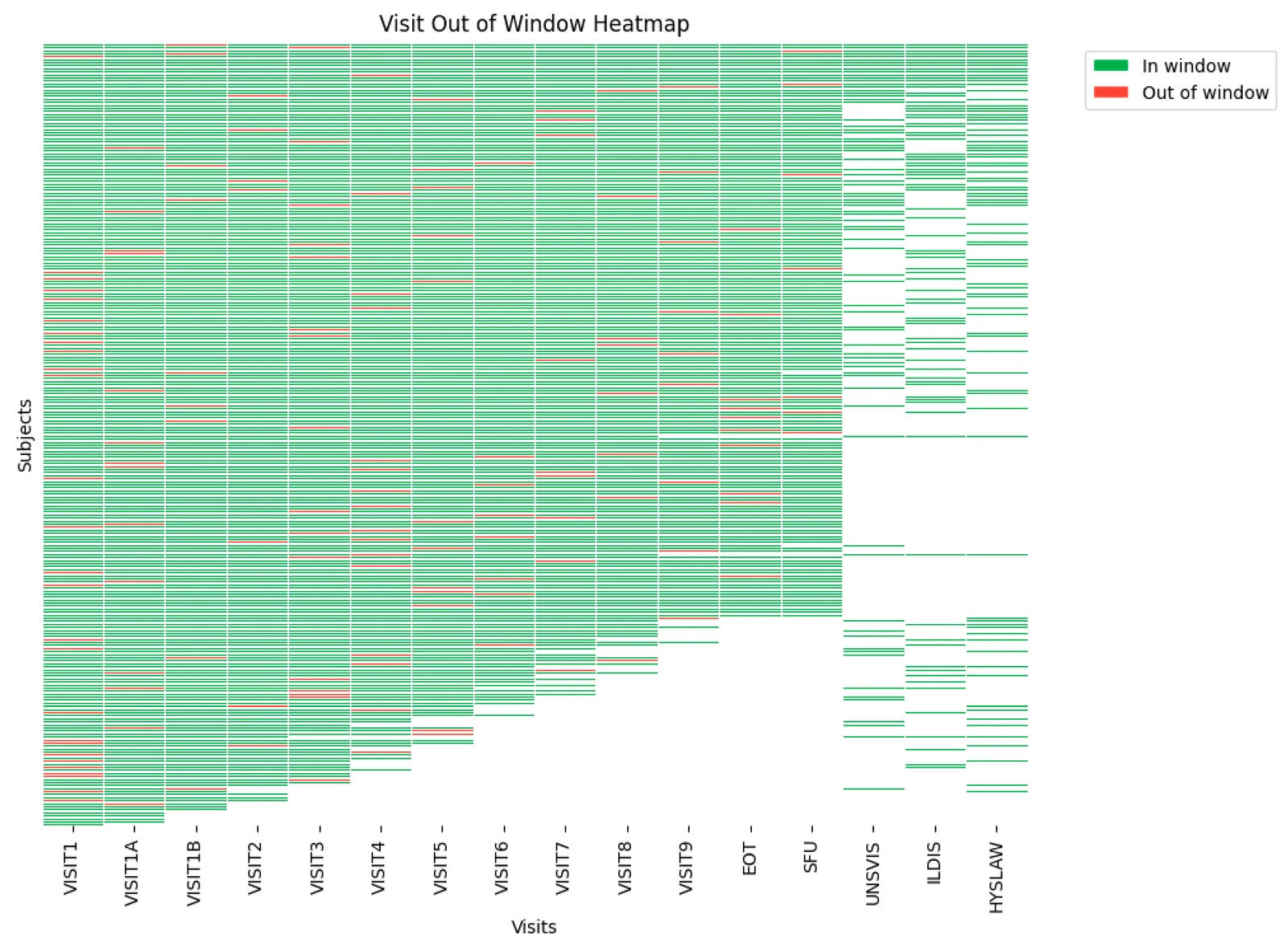
Example of a noise scenario in which a scheduled visit and its expected assessments are placed outside the permitted time window specified in the Visit Matrix (ALS). The modification introduces a controlled protocol deviation affecting visit timing and related records, enabling validation of visit-window checks, schedule compliance logic, and downstream reporting workflows.

**Figure 13:**
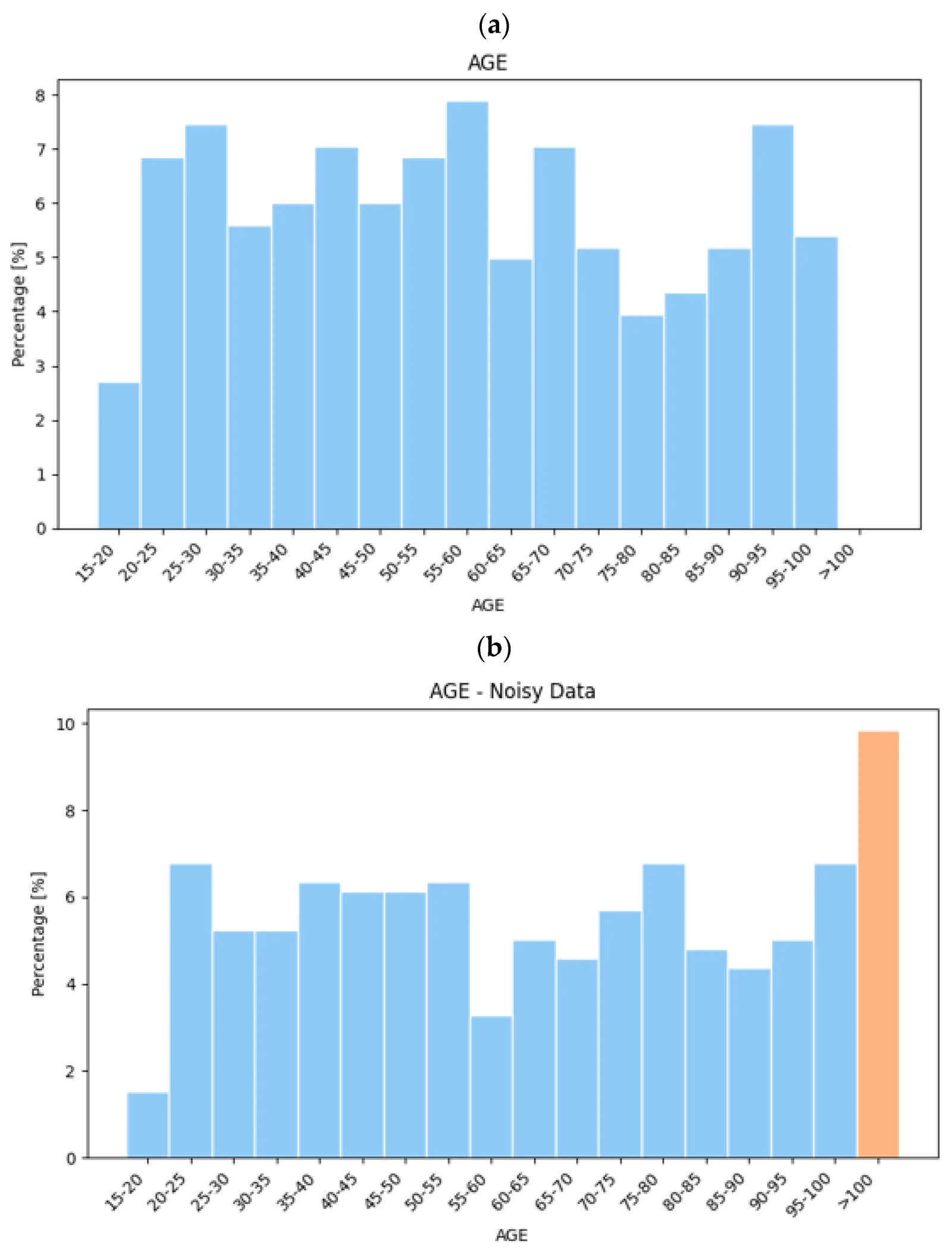
Example of a numeric-threshold error in which selected AGE values have been modified to exceed 100 years. (**a**) Distribution of AGE values before and after the threshold violation; (**b**) relationship between AGE and the affected record-level variable.

**Figure 14:**
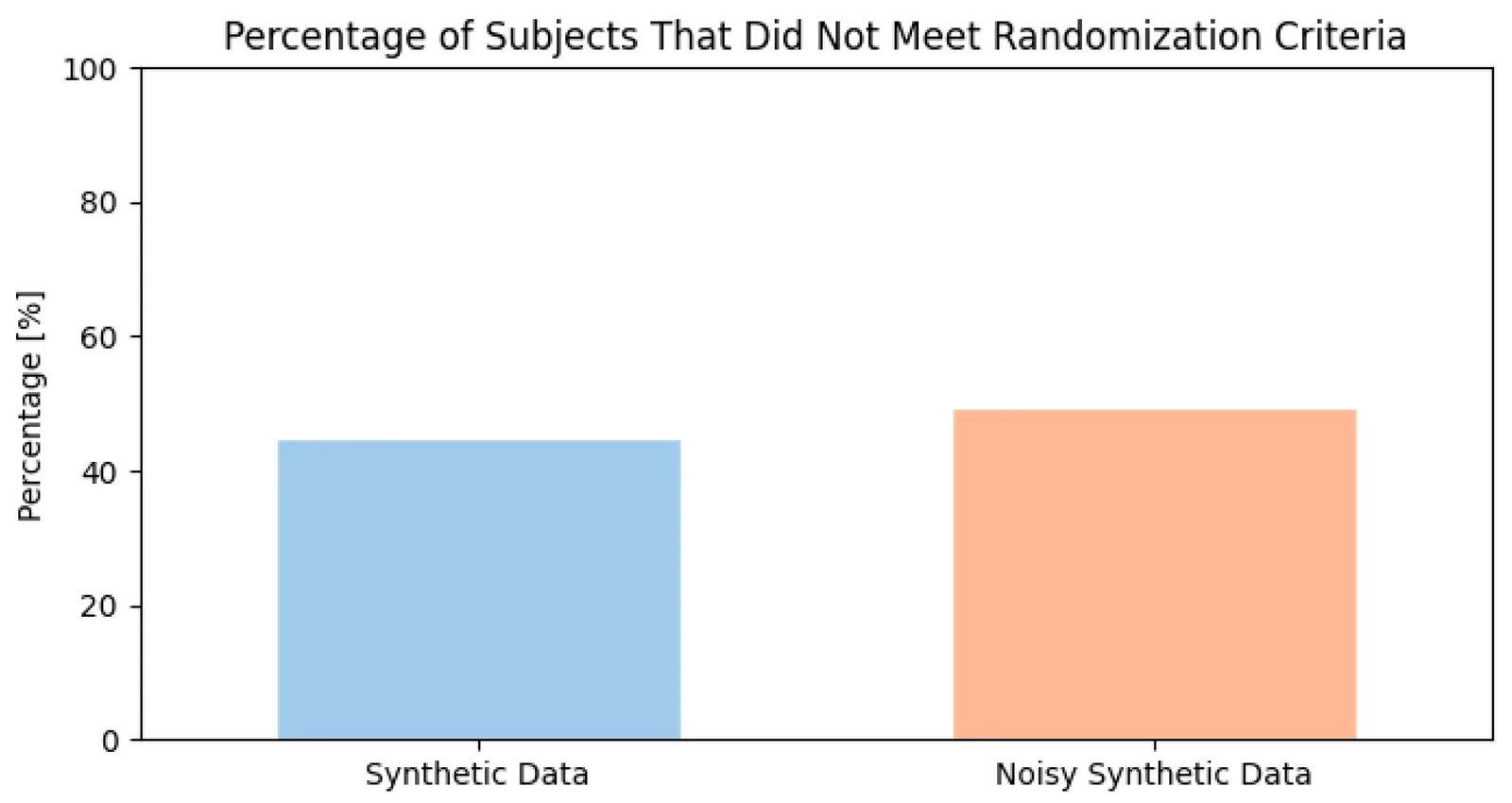
Example of a noise scenario introducing inconsistencies between randomization information (e.g., randomization date) and eligibility status (e.g., IE/IEYN), enabling validation of cross-form logic and protocol compliance rules.

**Figure 15:**
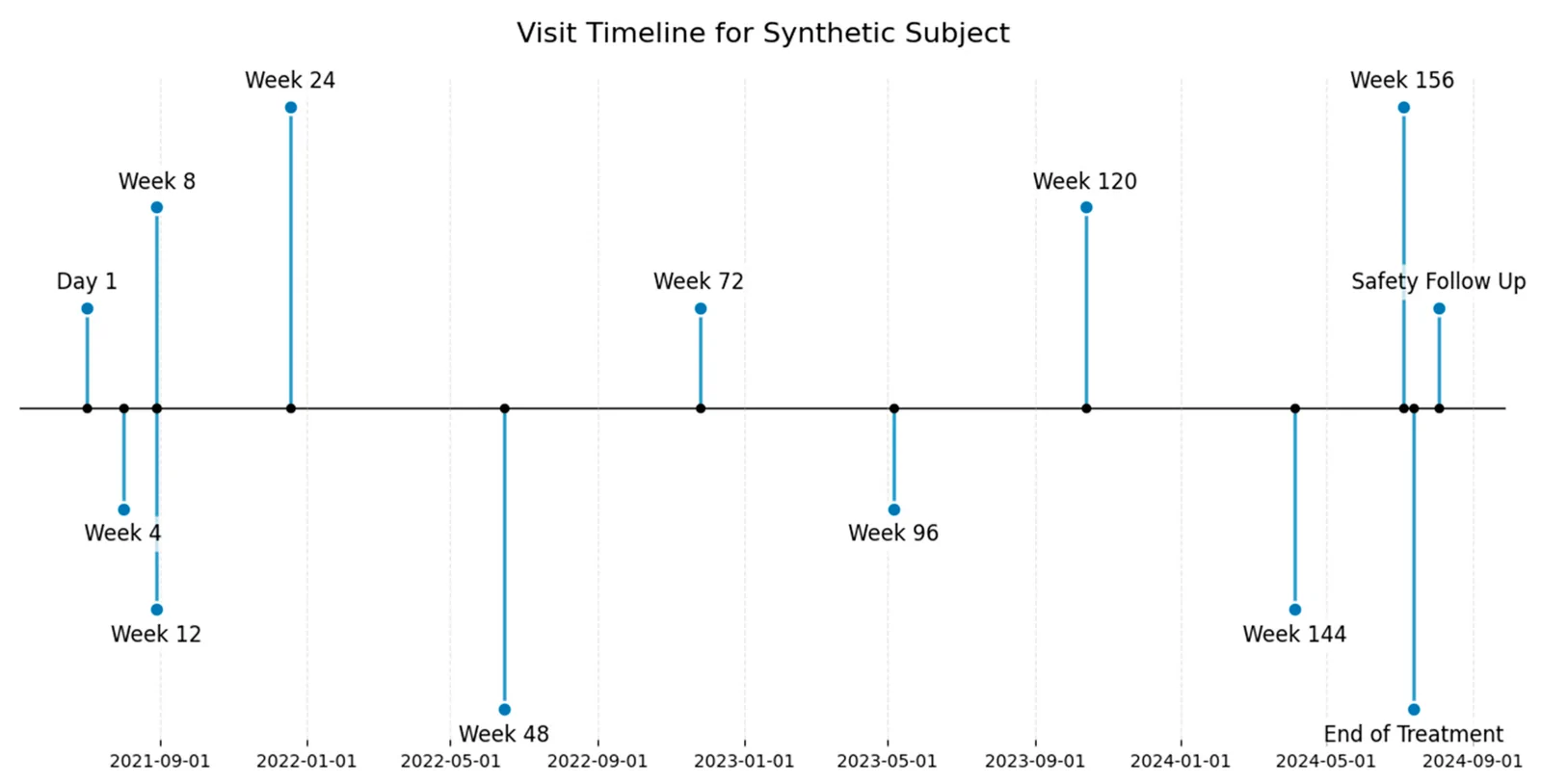
Example of a structural error in which two visits are assigned to the same calendar date, creating visit-date and potential key conflicts.

**Figure 16:**
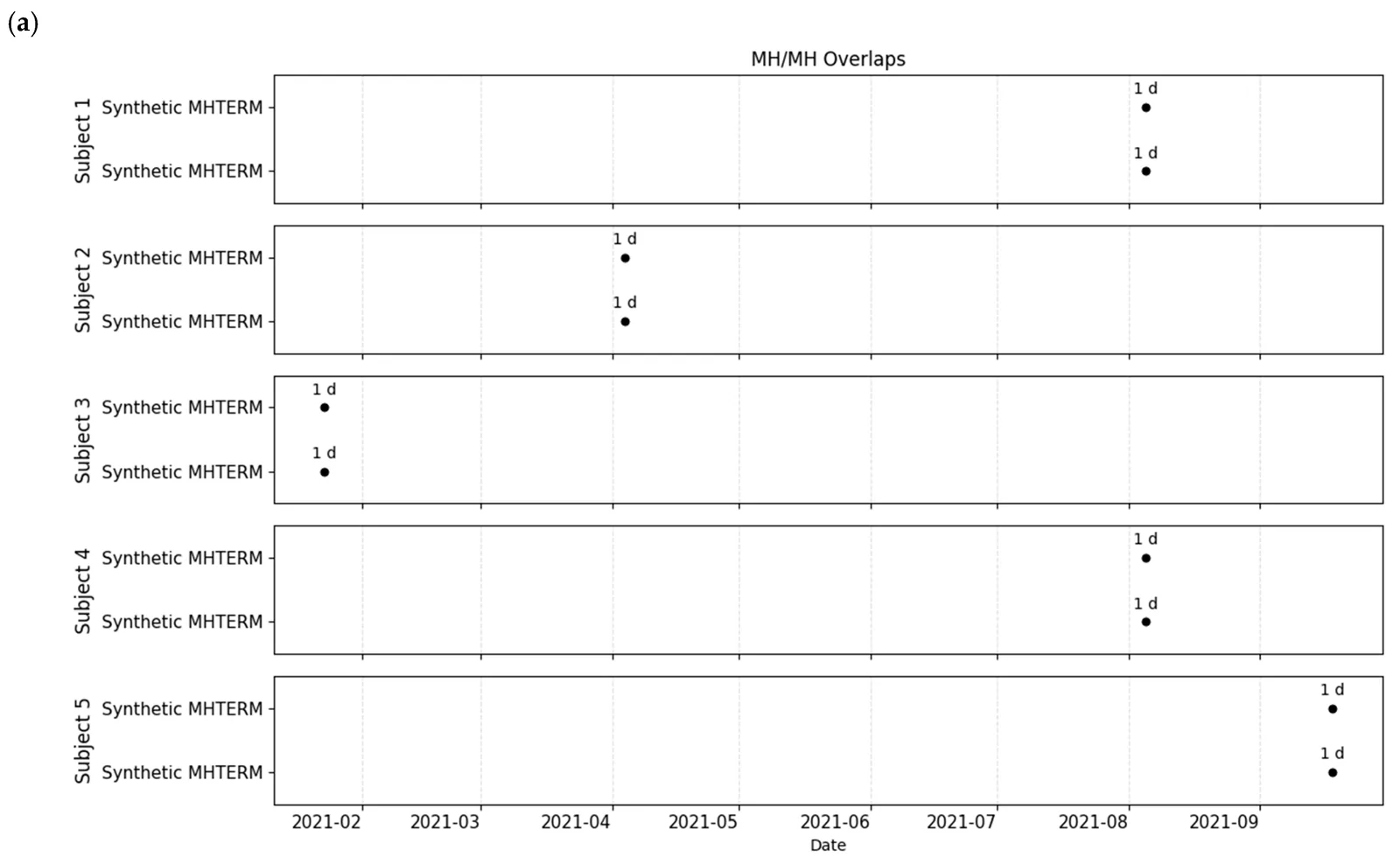

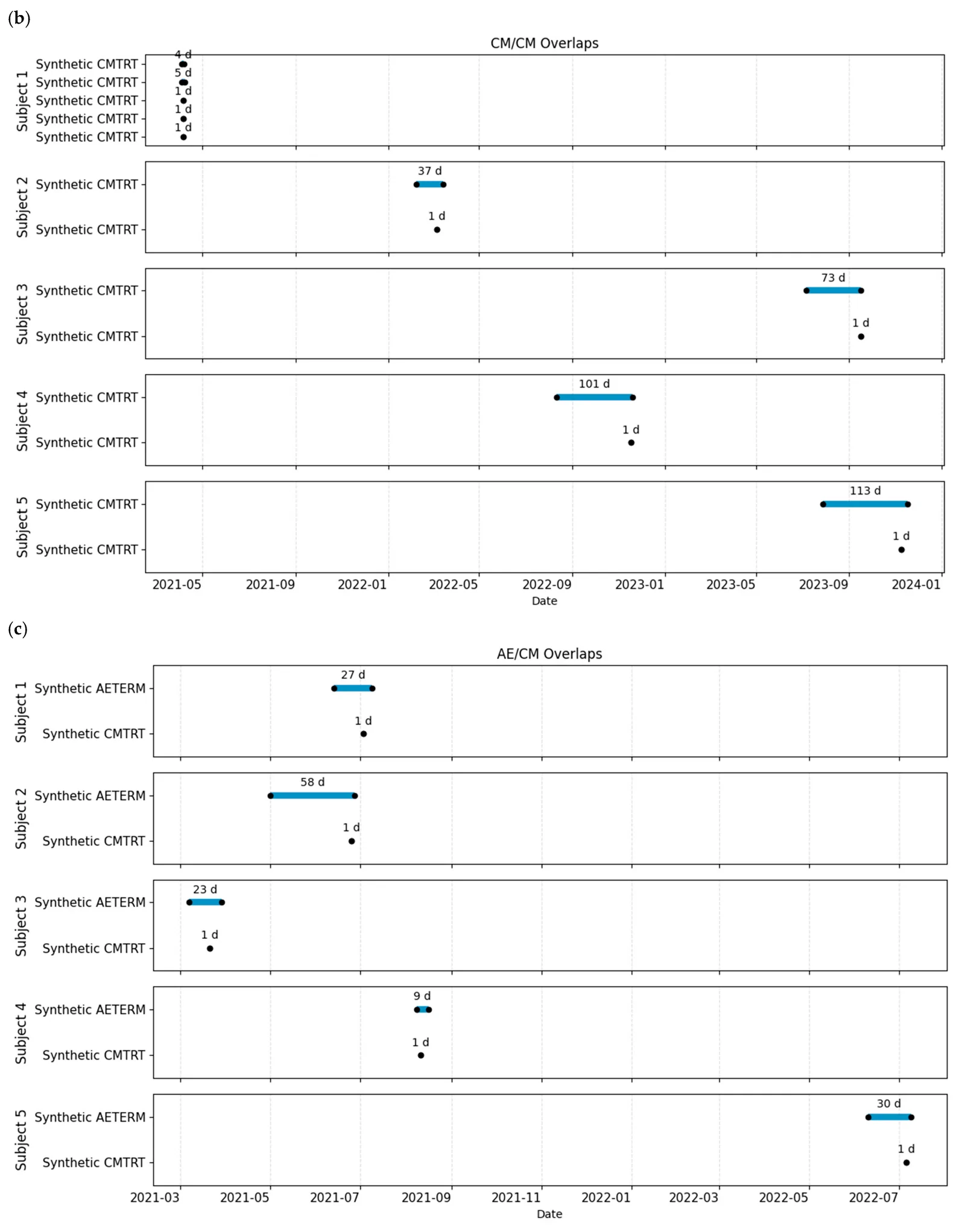

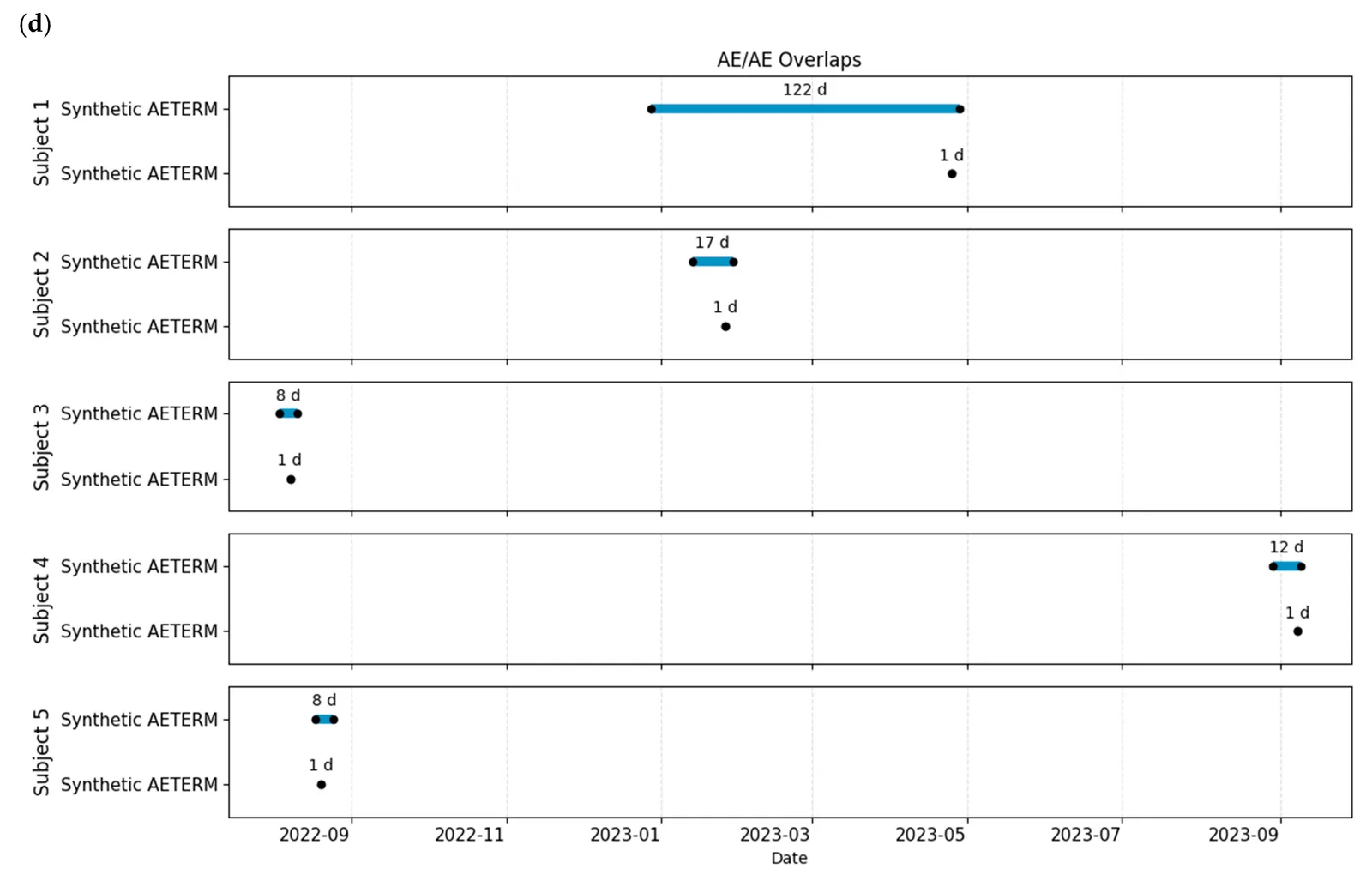
Four temporal-overlap patterns are generated as operational scenarios: (**a**) MH-MH, (**b**) CM-CM, (**c**) AE-CM, and (**d**) AE-AE. Temporal concurrency is not inherently an error. In particular, an AE-CM overlap does not establish a clinical-semantic link between a specific event and a medication.

We implemented 23 configurable error scenarios (Supplementary Table S1). Five representative classes are shown in Figure 11, Figure 12, Figure 13, Figure 14 and Figure 15: cross-dataset date inconsistencies, visit-window violations, numeric threshold exceedances, randomization or eligibility inconsistencies, and structural conflicts. These errors were deliberately injected into otherwise clean synthetic datasets with configurable intensities and deterministic seeding. Figure 16 is presented separately because temporal overlap is not inherently an error.

#### 3.2.1. Date Thresholds Across Datasets

An event date is modified to violate the minimum or maximum bounds defined in another dataset. Configuration: Target columns AESTDAT and DSSTDAT; five records modified; records joined by subject.

Figure 11 shows an example of a cross-dataset date inconsistency created by the Noise Tool, in which an event date is shifted to violate a bound defined in another dataset (e.g., an AE date occurring after discontinuation). This scenario is intended to stress-test cross-form timeline checks under controlled, reproducible conditions.

#### 3.2.2. Visit Outside the Permitted Window

A visit and its expected assessments have been shifted outside the window permitted by the ALS visit matrix. Configuration: 200 visit dates placed outside the allowed window.

Figure 12 illustrates a visit-window violation introduced by shifting a scheduled visit outside the allowed time window defined in the ALS visit matrix (Targetdays ± OverDueDays). Depending on the configuration, the shift can be applied at the level of an individual visit or propagated across the participant’s associated assessments for that visit, thereby creating realistic downstream inconsistencies. This scenario supports testing of visit scheduling and protocol compliance checks, as well as downstream derivations and reporting logic that rely on correct visit timing (e.g., window-based flags, baseline/endpoint selection, and visit-to-visit interval calculations). Because the perturbation frequency and random seed are controlled, the same violation patterns can be reproduced across runs to benchmark validation performance consistently.

#### 3.2.3. Result Range Thresholds

A numerical result is modified to exceed a specified threshold.

Configuration: Target column AGE; upper threshold: 100; 10% of records affected.

Figure 13 presents a numeric threshold violation in which AGE values have been modified to exceed a predefined validation threshold of 100 years. The scenario is intended to test whether range-checking and downstream validation logic correctly identify deliberately introduced out-of-range values.

#### 3.2.4. Incorrect Randomization

An inconsistency has been created between randomization information and eligibility status. Configuration: Target columns DSCSTDAT and IEYN; 10% of records affected.

IE/IEYN—Inclusion/Exclusion Criteria = Yes/No.

Figure 14 shows an intentionally introduced inconsistency between randomization information (e.g., randomization date) and eligibility status (e.g., IE/IEYN). In this scenario, records are modified so that a participant appears randomized despite failing eligibility, or conversely, remains marked as eligible without a corresponding valid randomization record. The purpose is to stress-test cross-form reconciliation logic and protocol compliance checks, including rules that validate temporal ordering (eligibility confirmation prior to randomization), consistency of subject status across modules, and downstream derivations that assume a coherent randomization pathway. By controlling the affected proportion of records and using deterministic seeding, the Noise Tool enables reproducible evaluation of whether validation pipelines reliably detect and flag such inconsistencies.

#### 3.2.5. Structural Conflict: Duplicate Lines or Two Visits on the Same Day

Duplicate rows are inserted, or two visits are assigned to the same calendar date for a patient. Configuration: ten patients with two visits on the same day.

Figure 15 demonstrates a structural conflict generated by the Noise Tool, which creates same-day visit collisions for the same participant. In the visit-collision variant, two nominally distinct visits are forced onto the same calendar date, creating inconsistent visit sequencing and potential key collisions across visit-dependent modules. This scenario is used to stress-test structural validation, including detection of duplicate keys, enforcement of uniqueness constraints, visit-order logic, and downstream handling of conflicting records during listing generation, aggregation, and reconciliation workflows. In another possible variant, Duplicated Lines, one or more rows are replicated using the same identifying keys (e.g., subject and event/visit identifiers), mimicking common operational issues such as accidental double entry, import retries, or reconciliation errors.

#### 3.2.6. Operational Temporal-Overlap Scenarios

Figure 16 shows reproducible within- and cross-domain temporal overlaps used to test interval handling and reporting workflows. These scenarios represent concurrent records and must be distinguished from deliberately erroneous overlaps or structural collisions. The present implementation does not infer whether an overlapping medication was prescribed for a specific adverse event.

## 4. Discussion

This study presents a modular framework for generating synthetic clinical trial datasets that conform to Medidata Rave ALS specifications while incorporating selected empirical patterns from a matched reference study. The framework separates schema conformance, driven by ALS-defined forms, fields, visit schedules, and operational rules, from statistical fidelity, defined here as descriptive agreement for selected distributions and dependencies. The strongest evidence concerns ALS/eCRF-conformant generation for validation, report development, dry runs, edit-check testing, and related operational workflows. The study does not establish formal statistical equivalence, clinical validity, privacy protection, or uniform fidelity across domains.

Agreement was variable-specific. The selected binary operational fields shown in Figure 4, Figure 5, Figure 6 and Figure 7 exhibited small observed discrepancies in category composition, whereas descriptive agreement was weaker for selected high-cardinality AE and CM variables evaluated elsewhere in the generated outputs. These findings should not be generalized across entire domains. In addition, record-level category composition must be interpreted separately from patient-level event burden, which was not assessed in the reported comparisons.

The continuous EGORRES display showed close descriptive agreement in the principal concentration regions; however, the study did not perform formal equivalence testing, variable-specific cross-validation, or a dedicated analysis of rare extremes. Applications that depend on threshold behavior or tail probabilities therefore require additional validation. The main results do not support conclusions about continuous laboratory analytes because Figure 6 evaluates the categorical LBPERF field.

Shifts in selected baseline characteristics, such as age, illustrate that schema conformance does not guarantee preservation of cohort composition. The current version does not generate clinically calibrated treatment- or control-arm trajectories for comparative longitudinal analysis. Such use would require a prespecified inferential objective and a substantially more complex conditional, history-dependent architecture.

A practical strength of the framework is its reproducible scenario engine and Noise Tool, which enable controlled testing of visit windows, conflicting dates, duplicated records, and other validation conditions. Operationally plausible temporal overlaps are distinguished from deliberately introduced errors. The reference dataset should be understood as an empirical benchmark for this fit-for-purpose evaluation rather than as a control group in a conventional comparative analysis.

Several limitations remain. First, synthesis quality depends on the representativeness, cleanliness, and transferability of the reference study. Second, Silverman’s rule was applied only to EGORRES evaluation; other KDE-based components were not reoptimized, and tail calibration was not assessed. Third, similarity was evaluated for selected variables and dependency structures, without repeated independent cohort generation or a complete patient-level record-burden analysis. Fourth, the conditional-missingness procedure does not identify or model MCAR, MAR, or MNAR mechanisms. Fifth, the framework has not been validated for multiyear disease trajectories, recurrent events, competing risks, or informative longitudinal dropout. Early termination and discontinuation are represented primarily through scenario rules rather than through joint history-dependent models.

Privacy was not evaluated empirically. Synthetic generation does not itself establish anonymization or the absence of disclosure risk, and the effects of preprocessing decisions or reduced cross-module linkage on disclosure risk were not assessed. Any use involving external release would require a prespecified threat model and a fit-for-purpose privacy evaluation, such as membership-inference, record-linkage, or nearest-neighbor analyses, as appropriate to the release setting.

Cross-module clinical-semantic relationships were not learned systematically. The implementation includes selected operational rules, such as serious adverse event pairing and temporal constraints, but temporal overlap does not establish that a medication was prescribed for a particular adverse event. Reduced preservation of fine-grained multitable trajectories limits analytical realism and should not be interpreted as evidence for improved privacy.

The generated datasets are therefore suited to schema validation, listings, edit-check testing, reporting development, and monitoring workflow preparation. Analyses that depend on causal interpretation, treatment-event-medication pathways, calibrated longitudinal outcomes, or patient-level event burden require additional modeling and validation. Figure 9 demonstrates how selected within-module categorical dependencies are encoded, but it does not establish comprehensive clinical coherence.

## 5. Conclusions

The results support SYNDATA as a framework for reproducible ALS/eCRF-conformant data generation for operational validation, dry runs, reporting development, edit-check testing, and workflow preparation before real study data are available. The study demonstrates close descriptive agreement for selected variables, but not uniform statistical fidelity, formal equivalence, clinical validity, or privacy protection. More complex safety and medication variables and cross-module clinical relationships require further development.

More analytically interpretive applications would require patient-level and variable-specific validation, stronger cross-domain linkage, uncertainty assessment across repeated generations, and privacy evaluation matched to the intended use and release model. Broader reuse across studies and therapeutic areas remains an implementation objective rather than a conclusion established by this evaluation.

## Figures and Tables

**Table 1:** Conceptual positioning of SYNDATA relative to major families of synthetic-data approaches. The entries summarize typical design emphases rather than capabilities that are universally present or absent in each methodological family. The comparison is conceptual and should not be interpreted as evidence of comparative performance or methodological superiority.

Dimension	SYNDATA	Deep Generative Approaches	Conventional Statistical Synthesis
Primary objective	Operational validation and workflow testing	Statistical/task-based fidelity	Reproduction of specified statistical structure
Native ALS/eCRF conformance	Core design feature	Typically requires adaptation or conditioning	Requires custom adaptation
Visit-schedule logic	Explicitly encoded	Typically requires conditioning or postprocessing	Requires custom rules
Configurable fault injection	Core design feature	Not typically a primary design objective	Not typically a primary design objective
Cross-domain clinical coherence	Limited; selected rule-based links	Potentially learnable; data-dependent	Model- and rule-dependent
Formal privacy guarantee	Not provided	Not inherent	Not inherent
Evidence provided in this study	Fit-for-purpose evaluation	Not benchmarked	Not benchmarked

**Table 2:** Distributional discrepancy metrics for selected categorical variables shown in the main figures, ranked by Jensen–Shannon distance. AE, adverse events; EG, ECG test results; MH, medical history; VISIT, visit dates; LB, laboratory test results. Lower values indicate closer descriptive agreement. No universal equivalence or acceptability threshold was applied. Comparisons were performed at the record level and do not establish similarity in patient-level event burdens.

Module	Variable	Synthetic Count	Source Count	Categories	Cramér’s V (95% Bootstrap CI)	JS Distance (95% Bootstrap CI)
EG	EGTEST	19,445	17,520	5	0.0000 (0.0000, 0.0137)	0.0000 (0.0009, 0.0062)
	EGORRES	19,445	17,580	2	0.0000 (0.0000, 0.0101)	0.0000 (0.0001, 0.0082)
MH	MHONGO	894	2550	2	0.0000 (0.0000, 0.0383)	0.0041 (0.0005, 0.0318)
	MHCURM	894	2345	2	0.0000 (0.0000, 0.0376)	0.0052 (0.0004, 0.0348)
VISIT	VECNTMOD	2628	4293	3	0.7583 (0.7451, 0.7700)	0.5889 (0.5790, 0.5992)
LB	LBPERF	4684	3934	2	0.0000 (0.0000, 0.0277)	0.0064 (0.0003, 0.0212)
AE	AECONTRT	477	2912	2	0.0241 (0.0000, 0.0592)	0.0301 (0.0030, 0.0634)
	AEANY	630	3770	2	0.0000 (0.0000, 0.0409)	0.0116 (0.0006, 0.0401)

## Data Availability

The reference (source) clinical trial data and code are not publicly available due to confidentiality restrictions and internal data governance requirements. Synthetic outputs may be made available upon reasonable request, subject to governance review and approval.

## Supplementary Materials

The following supporting information can be downloaded at: https://www.mdpi.com/article/10.3390/healthcare14172870/s1. Table S1: Noise-induced error scenarios implemented in the SYNDATA framework.

## Abbreviations

The following abbreviations are used in this manuscript:

ALS	Architect Loader Spreadsheet
eCRF	Electronic Case Report Form
AE	Adverse Event
CM	Concomitant Medication
AENO	Adverse event number (primary counter for AE)
AEOUT	Adverse Event Outcome
AEPRR	AE Required Procedure
AECONTRT	Concomitant of Additional Treatment Given
AEACN	Action Taken, Investigational Product
AECAT	Category for Adverse Event
AECATOCC	AE Category Occurrence,
AETOXGRM	Maximum CTCAE Grade
AESER	Serious Adverse Event
AEREL	Reasonable Possibility AE Caused by IP
CAT	Suffix indicating a categorical field/categorical-coded field (used to prevent cross-population when column name ends in “CAT”)
EG	ECG Test Result
EGORRESU	ECG Test Unit
EGTEST	ECG Test Name
EOT	End of Treatment
IE	Inclusion/Exclusion Criteria
IEYN	Inclusion/Exclusion Criteria Met Yes/No
KDE	Kernel density estimation
LB	Laboratory Test Result
MedDRA	Medical Dictionary for Regulatory Activities (named as an external coding dictionary)
MH	Medical history
RANNDAT	Randomization Date
SCR	Screening Visit
SERAE	Serious adverse event report
SFU	Survival Follow up Period
SME	Subject matter expert
SYNDATA	Name of the modular synthetic data generation framework/tool described
WHODrug	WHO Drug Dictionary (named as an external coding dictionary)
