# Supplementary material for: Leveraging Synthetic Clinical Data for Validation and Operational Readiness in Clinical Trials
Source: Healthcare (Basel). 2026 Sep 7;14(17):2870. doi: 10.3390/healthcare14172870 (PMC13565869; doi:10.3390/healthcare14172870)
Supplement: Supplementary file 1 [file healthcare-14-02870-s001.zip › healthcare-4454036-supplementary.pdf]

Supplementary Table – S1 – Noise-induced error scenarios implemented in the SYNDATA framework.

No	Scenario	Description
1	Missing Data	Introduce missing value in specified column (formatted as per ALS)
2	Minimum Code Length	Truncate a value to make it shorter than a specified expected length (as per ALS)
3	Maximum Code Length	Extend value to make it longer than a specified maximum length (as per ALS)
4	Wrong Character in Code Numeric	Modify value in a numeric column to contain prohibited characters
5	Date Format	Change the date format to not match the one specified in that field (as per ALS)
6	Date Threshold Value	Change date in a column to make it earlier or later than a specified min or max date threshold provided by end user
7a	Date Threshold – Same Dataset	Change date in a column to make it larger or smaller than a specified min or max date threshold stored in another column in the same dataset
7b	Date Threshold – Different Datasets	Change date in a column to make it larger or smaller than a specified min or max date threshold stored in another column in another dataset
8	Result Range Above Threshold	Change numeric value in a column to make it larger than a specified max numeric threshold provided by end user
9	Result Range Below Threshold	Change numeric value in a column to make it smaller than a specified numeric threshold provided by end user
10	Incorrect Time Format	Change the time format to not match the one specified in that field (as per ALS)
11	Incorrect Randomization	RANDDAT present but IE/IEYN = No for the same patient and screening and/or randomization visit
12	Rescreening Outside of Window	Rescreening visits occurring earlier than specified (as per ALS)
13	Rescreening Outside of Range	Rescreening visits occurring more times than specified (as per ALS)
14	Visit Outside of Window	Visit and expected assessment outside of range specified in Visit Matrix
15	Missing Visit	Visit and expected assessments missing for a random visit according to Visit Matrix.
16	Random Missing DataPoints	For datasets expected on each visit per visit matrix, remove randomly some datapoints

17	Inactivated Loglines	Insert selected number of inactivated rows in selected datasets
18	Duplicated Lines	Insert same values for same patient and same dataset
19	Two Visits Same Day	Insert two visits the same day for the same patient
20	AE/AE Overlap	Introduce two Adverse Events overlapping in time
21	AE/CM Overlap	Introduce Adverse Event and Concomitant Medication intake overlapping in time
22	CM/CM Overlap	Introduce two Concomitant Medication intakes overlapping in time
23	MH/MH Overlap	Introduce two Medical History events overlapping in time
